# Identification and Mechanisms of Osteocyte Subsets Involved in the Pathological Progression of Osteoporosis

**DOI:** 10.1002/advs.202513427

**Published:** 2025-11-18

**Authors:** Zengxin Jiang, Biyu Rui, Xuecheng He, Jiajun Wang, Xiangdong Wu, Rui Shao, Xue Meng, Xiaochun Peng, Hengfeng Yuan

**Affiliations:** ^1^ Department of Orthopaedics Shanghai Jiaotong University Affiliated Sixth People's Hospital No. 600 Yishan Road Shanghai 200233 China; ^2^ Institute of Microsurgery on Extremities Shanghai JiaoTong University Affiliated Sixth People's Hospital Shanghai 200233 China; ^3^ Department of Orthopaedics Beijing Jishuitan Hospital Capital Medical University Beijing 100035 China

**Keywords:** bone homeostasis, osteocyte subsets, osteoporosis, Sema5a

## Abstract

Osteocytes are the most abundant cell type in bone. However, the detailed functions of osteocyte subsets in osteoporosis have remained obscure. In this study, it is aimed to investigate the impact of osteocyte subset heterogeneity on the pathological process of osteoporosis and new potential molecular mechanisms. Six osteocyte subsets are identified in mouse bones by single‐cell sequencing. Among them, the epidermal growth factor receptor (Egfr)+ interleukin‐1 receptor type I (Il1r1)+ Semaphorin 5a (Sema5a)+osteocyte subpopulation (bone homeostasis regulatory osteocytes, BHR‐Ocys) played a key role in the maintenance of bone homeostasis by regulating osteoblasts and osteoclasts. The data confirm that the increased proportion of BHR‐Ocys contributes to bone loss in the postmenopausal osteoporotic mice. Mechanistically, Sema5a derived from BHR‐Ocys binds to the osteoclast receptor protein Plexin A1 (Plxna1), thereby activating the phosphatidylinositol‐3 kinase/protein kinase B/myelocytomatosis oncogen (PI3K/AKT/Myc) signaling pathway, leading to active osteoclast‐mediated bone resorption. In conclusion, BHR‐Ocys is a key cell subpopulation in the regulation of bone homeostasis. The Sema5a‐Plxna1 axis is an important mechanism for BHR‐Ocys to regulate osteoclast differentiation. The study provides new insights into the role and mechanism of osteocytes in the regulation of bone homeostasis and identifies new targets for the prevention and treatment of osteoporosis.

## Graphical abstract

Osteocyte heterogeneity in bone remains poorly understood. This study identifies six distinct osteocyte subsets in mice, revealing a key subpopulation (BHR‐Ocys) that drives bone loss in osteoporosis. BHR‐Ocys promote osteoclast‐mediated resorption via a novel Sema5a‐Plxna1 signaling axis. This research unveils new mechanisms regulating bone homeostasis and identifies promising therapeutic targets for treating osteoporosis.

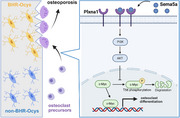

## 1 Introduction

Osteoporosis is a common disease characterized by decreased bone density and an elevated risk of fractures. The imbalance of bone remodeling serves as the primary pathogenesis of osteoporosis.^[^ ^1^ ^]^ Bone tissue is a dynamic biological structure whose formation and remodeling depend on the interactions among various cell types. Osteocytes represent the most abundant cell type within bone tissue, playing a crucial role in regulating the balance of bone metabolism through the secretion of diverse signaling molecules.^[^ ^2^, ^3^, ^4^, ^5^ ^]^ The dysfunction of osteocytes is recognized as a significant contributor to the pathogenesis of osteoporosis.^[^ ^6^, ^7^ ^]^

Cell subpopulations refer to distinct groups within the same cell type that exhibit heterogeneity in morphology, function, or gene expression.^[^ ^8^ ^]^ Recent advancements in single‐cell RNA sequencing technology have enabled deeper insights into the gene expression profiles of different cell subpopulations, elucidating their roles in various physiological and pathological conditions.^[^ ^9^ ^]^ A comprehensive understanding of cell subpopulations facilitates the uncovering of disease mechanisms and may offer a foundation for the development of new therapeutic strategies.^[^ ^10^ ^]^ For instance, different chondrocyte subpopulations display unique functions at various stages of osteoarthritis.^[^ ^11^ ^]^ It is reasonable to speculate that distinct subpopulations of osteocytes play specific roles in the onset and progression of osteoporosis. Investigating specific osteocyte subpopulations will enhance our understanding of osteocyte functionality and their involvement in osteoporosis, potentially uncovering novel cellular and molecular mechanisms and providing new avenues for treatment in the bone‐related diseases. Nevertheless, the heterogeneity among osteocytes remains poorly understood.

In this study, we found a new population of osteocytes characterized by epidermal growth factor receptor (Egfr)+ interleukin‐1 receptor type I (Il1r1)+ Semaphorin 5a (Sema5a)+ in mice through single‐cell sequencing. The Egfr+ Il1r1+ Sema5a+ osteocyte subpopulation served as a primary source of osteogenic and osteoclastic regulatory factors, such as receptor activator of nuclear factor‐κ B ligand (RANKL) and secreted phosphoprotein 1 (SPP1), and played a pivotal role in maintaining bone homeostasis. We thus identified this new osteocyte subpopulation as bone homeostasis regulatory osteocytes (BHR‐Ocys).

Sema5a, a member of the semaphorin family, is a secreted and membrane bound glycoprotein that plays a crucial role in tissue development and organism health.^[^ ^12^, ^13^ ^]^ However, the specific role of Sema5a in bone metabolism is unclear. We found that BHR‐Ocys could modulate the differentiation and bone resorption function of osteoclasts by secreting Sema5a which interacts with Plxna1 receptor on osteoclasts. Further investigation suggested that Sema5a‐Plxna1 axis was a key upstream regulator of Myc‐mediated osteoclastogenesis and pathological bone resorption, which representing a crucial mechanism in the onset and progression of osteoporosis. Our study offered new insights into potential cellular and molecular pathways involved in the osteoporosis and shed light on new directions of osteoporosis treatments.

## 2 Results

### 2.1 Identification of Subgroups of Osteocytes within the Bone

**Figure 1:**
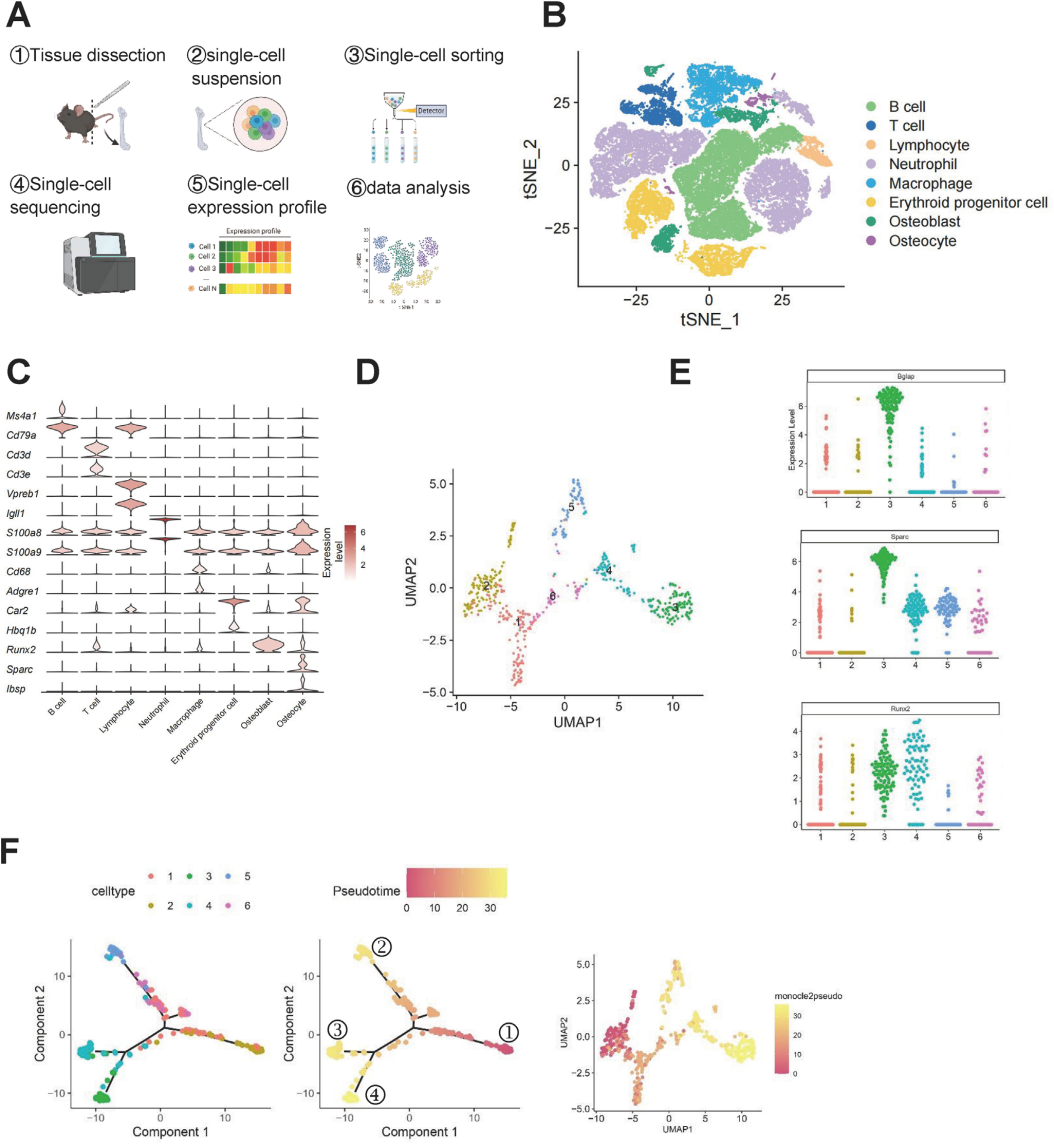
The analysis of osteocyte subsets by single‐cell sequencing of mouse femurs. A) Schematic workflow of the experimental strategy. B) t‐SNE dimensional reduction visualizations 8 cell types. C) Violin plot showing the expression of specific signatures in identified cell types. D) UMAP analysis of 6 osteocyte group subsets. E) Dot plot showing the expression level of Bglap, Runx2, and Sparc in each osteocyte subcluster. F) Monocle pseudotime trajectory analysis of the six osteocyte subsets.

We collected femoral bone tissue from the sham surgery and ovariectomy (OVX) groups of mice for single‐cell sequencing, as illustrated in **Figure** **1**A. All cells were annotated based on the expression of enrichment genes in each cluster. Specifically, eight clusters were identified: (1) osteocytes (Sparc/Ibsp), (2) osteoblasts (Runx2), (3) macrophages (Cd68/Adgre1), (4) T cells (Cd3d/Cd3e), (5) B cells (Ms4a1/Cd79a), (6) lymphocytes (Vpreb1/Igll1), (7) neutrophils (S100a8/S100a9), and (8) erythroid progenitor cells (Car2/Hbq1b) (Figure 1B,C). Osteocytes were the primary focus in our study. Subgroup analysis revealed that the osteocytes could be divided into 8 subclusters (C0‐C7, 701 cells in total, Figure S1A, Supporting Information). Heat map represents Pearson correlations of 8 osteocyte subsets with each other (Figure S1B, Supporting Information). To exclude potentially contaminating cells of other types, we further analyzed the marker genes of each subpopulation. The results revealed that clusters C0 and C7 exhibited high similarity and both highly expressed erythroid‐specific marker genes, including Gata1, Klf1, Car1, and Car2 (Figure S1C,D, Supporting Information). These clusters were therefore considered likely to represent contaminating erythroid precursors introduced during the clustering process and were subsequently excluded. The remaining six clusters (C1–C6) of bone cells were retained for subsequent analysis (Figure 1D). To ensure that the cells included in the analysis were osteocytes, we further incorporated additional well‐established markers, including Sp7, Col1a1, Bglap2, and Dmp1, in the clustering and visualization analyses. Significantly higher expression of Sp7, Col1a1, Bglap2, and Dmp1 were observed in osteocytes compared to other cell populations, thus confirming the osteocytic identity of the analyzed cells (Figure S2, Supporting Information).

Characteristic osteocyte genes such as Bglap, Runx2, and Sparc are illustrated in the expression profiles of each subcluster shown in Figure 1E. The pseudotime analysis of the six osteocyte subclusters is presented in Figure 1F. Three major clades were found. The osteocytes in C1 were the roots of differentiation (number 1), and the Osteocytes in C3, C4, and C5 were in the end‐point states (number 2–4).

**Figure 2:**
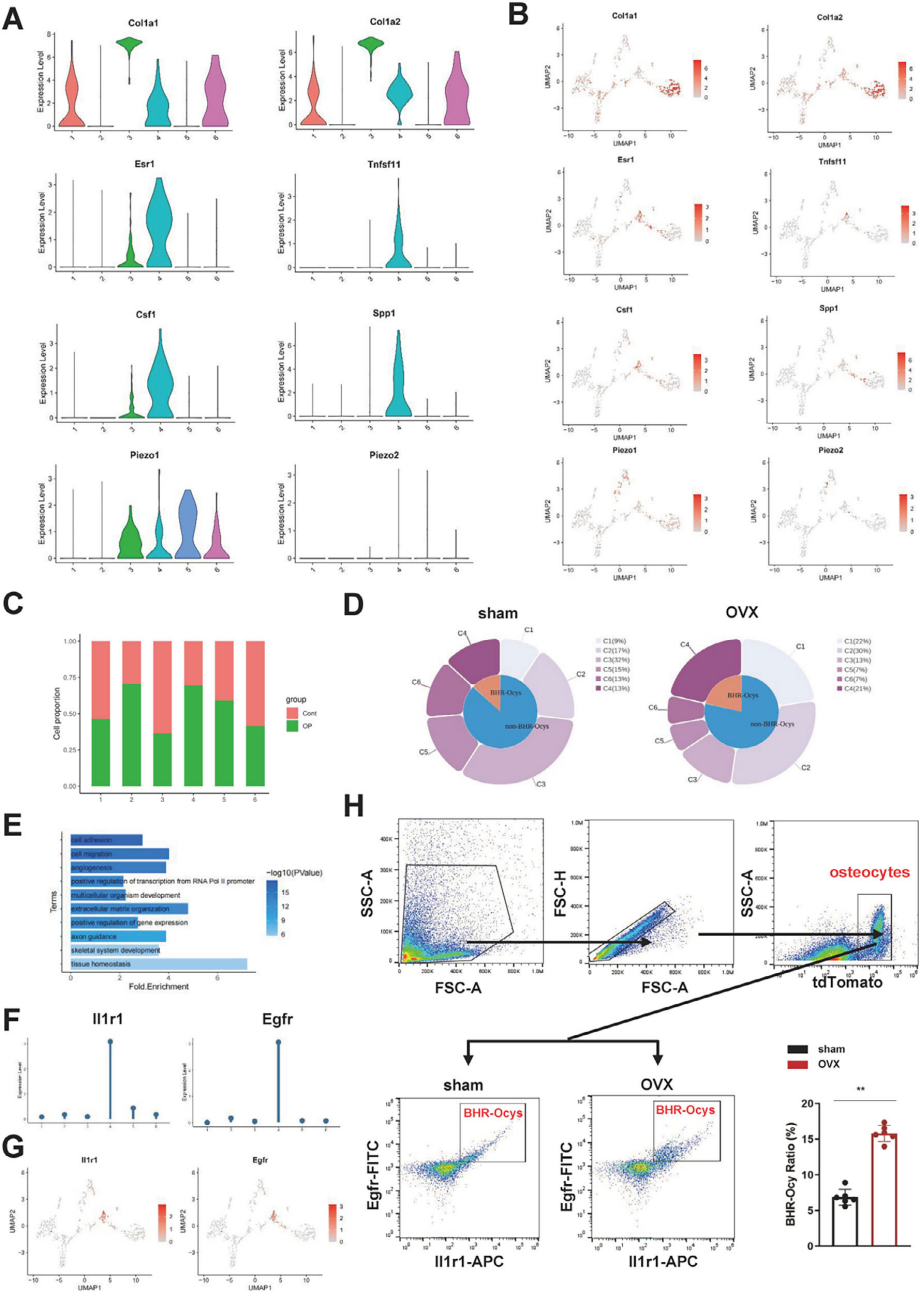
Identification of the BHR‐Ocy subpopulation. A) Violin plots showing the expression level of Tnfsf11, Csf1, Spp1, Esr1, Col1a1, Col1a2, Col5a2, Piezo1, and Piezo2 in each osteocyte subcluster. B) Feature plots showing the expression level of Tnfsf11, Csf1, Spp1, Esr1, Col1a1, Col1a2, Col5a2, Piezo1, and Piezo2 in each osteocyte subcluster. C) The bar plot showing the ratio of each subpopulation in the osteocytes from each condition. D) The circle plot representing the proportions of various osteocytes subpopulations in sham and OVX groups. E) Part of the GO analysis results based on DEGs between the BHR‐Ocys and other osteocyte subsets. F) Lollipop plots displaying the expression level of Egfr and Il1r1 in each osteocyte subcluster. G) Feature plots of Egfr and Il1r1 in osteocytes. H) Flow cytometry sorting of BHR‐Ocys (tdTomato+ Egfr+ Il1r1+ osteocytes) from sham and OVX mice femurs. The statistical analysis showing the ratios of BHR‐Ocy in sham and OVX mice (*n* = 6 per group). Data are represented as the mean ± SD. Statistical analysis employed two‐tailed unpaired Student's *t*‐test H). *p < 0.05, **p < 0.01.

We analyzed the expression levels of genes known to be crucial for osteocyte functions in each subcluster, including matrix synthesis‐related Col1a1, Col1a2; bone homeostasis‐regulating genes Tnfsf11, Csf1, Spp1; and mechanosensitive genes Piezo1 and Piezo2. Given the use of the OVX model, we also assessed the expression of Esr1 (**Figure** **2**A,B). Based on the above results, we formulated preliminary definitions of the functions of each osteocyte subcluster: C1‐Osteocytes involved in signal transduction, regulation, and response processes; C2‐Osteocytes associated with cytoskeletal remodeling; C3‐Osteocytes relevant to matrix synthesis; C4‐Osteocytes responsible for regulating bone homeostasis; C5‐Osteocytes participating in angiogenesis; C6‐Immunoregulatory Osteocytes. Col1a1, and Col1a2 were significantly upregulated in C3, further confirming the association between C3 and matrix synthesis. Tnfsf11 and Csf1 are key cytokines through which osteocytes regulate osteoclasts, while Spp1 play critical roles in osteoblast regulation. Tnfsf11, Csf1, and Spp1 were significantly upregulated in C4, which was linked to bone homeostasis regulation, thus reaffirming the osteocytes' vital role. Interestingly, Esr1 was significantly upregulated in C4, indicating that this subcluster was most affected by decreased estrogen levels. Notably, mechanosensitive Piezo1 is highly expressed in C3 to C7, while Piezo2 is relatively more abundant in C4 and C5.

The ratio of each subpopulation in the osteocytes from each condition were illustrated in Figure 2C. The circle plot representing the proportions of various osteocytes subpopulations in sham and OVX groups were illustrated in Figure 2D. To determine the primary functions of each osteocyte subcluster, we conducted the Gene Ontology (GO) enrichment analysis on their characteristic genes (Figure 2E; Figure S3, Supporting Information). Given the crucial role of C4 in regulating bone homeostasis, it was likely a key osteocyte subcluster influencing the onset and development of osteoporosis. Therefore, we designated C4 as BHR‐Ocys and concentrated on this subcluster throughout the study. To isolate BHR‐Ocys from all osteocytes, we identified cell surface proteins that were highly expressed in BHR‐Ocys and found that Egfr and Il1r1 were significantly upregulated in BHR‐Ocys (Figure 2F,G). We constructed transgenic mice expressing tdTomato in osteocytes (Dmp1‐Cre+; tdTomato+/+) and established the OVX model. The schematic representation of *the strategy* for constructing Dmp1‐Cre mice was shown in Figure S4A (Supporting Information). Red fluorescence was only observed in osteocytes embedded within the bone matrix, which verified the osteocyte‐specific expression pattern of Dmp1‐Cre in the current study (Figure S4B, Supporting Information). Furthermore, three potential off‐target site with higher scores (high off‐target risk) was amplified by PCR followed by sequencing. The information of off‐target sites was provided in Supplementary File 2. Full sequencing data are available in Supplementary File 3 provided with this paper. No off‐target sites were found (Figure S4C, Supplementary File 3, Supporting Information). The results of immunofluorescence co‐staining confirmed the existence of BHR‐Ocys (Figure S5, Supporting Information). Subsequently, flow cytometry was employed to isolate BHR‐Ocys (tdTomato+ Egfr+ Il1r1+ osteocytes). To verify the isolated cell types, we performed ex vivo culture of isolated cells. Images of cells on day 1, day 3, and day 7 after complete attachment are shown in Figure S6A (Supporting Information). Cells did not proliferate during cultivation. The absence of cell proliferation is consistent with the characteristic of osteocytes as terminally differentiated cells. We also observed that the isolated cells exhibited the characteristic dendritic morphology of osteocytes under scanning electron microscopy (Figure S6B, Supporting Information). In addition, we evaluated expression of SOST and Dmp1. The results showed that the expression levels of SOST and Dmp1 in the target cells isolated via flow cytometry sorting were significantly higher than in other cells (Figure S6C, Supporting Information). The results above collectively demonstrated the reliability of our osteocyte isolation method for research purposes. We found that the proportion of BHR‐Ocys in OVX mice was significantly increased compared to the sham group, which aligns with the cell proportion data presented in Figure 2C,D, suggesting that the upregulation of BHR‐Ocys may be associated with an imbalance of bone homeostasis in osteoporosis (Figure 2H).

### 2.2 BHR‐Ocys‐Derived Sema5a Promoted Osteoclast Differentiation

**Figure 3:**
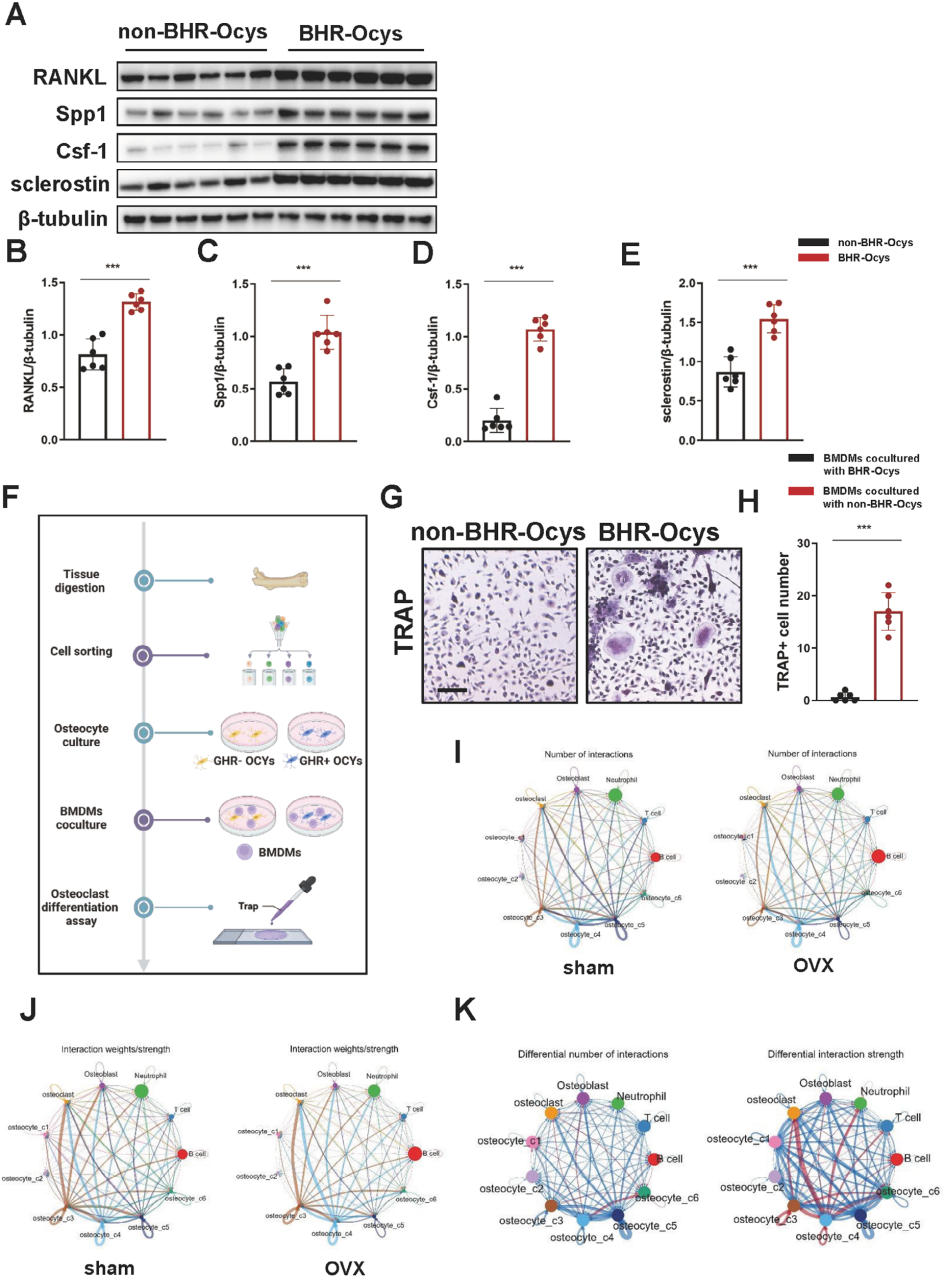
BHR‐Ocys maintained bone homeostasis by regulating osteoclasts. Representative Western blot of RANKL, Spp1, and Csf‐1 in BHR‐Ocys from OVX mice (each sample consisted of BHR‐Ocys isolated from the femurs and tibias of 10 mice, with six samples per group). Quantification of the protein level of B) RANKL, C) Spp1, D) Csf‐1, and E) sclerostin (*n* = 6 per group). F) Schematic workflow of the osteocyte‐osteoclast coculture experiment. G) TRAP staining of osteoclasts cocultured with BHR‐Ocys and non‐BHR‐Ocys (scale bar, 200 µm). H) Quantification of TRAP‐positive multinucleated (≥3 nuclei) cell number per well (*n* = 3). I) Circle plots showing the number of interactions between different osteocyte subclusters and other cell types in femurs of sham and OVX mice. J) Circle plots showing the strengths of interactions between different osteocyte subclusters and other cell types in femurs of sham and OVX mice. K) Circle plots showing the differential number and strengths of interactions between different osteocyte subclusters and other cell types in bone.Data are represented as the mean ± SD. Statistical analysis employed two‐tailed unpaired Student's *t*‐test (H). *p < 0.05, **p < 0.01.

We identified a group of osteocyte subpopulation involved in the regulation of bone homeostasis, designated as BHR‐Ocys. Utilizing flow cytometry, we isolated Egfr+ Il1r1+ Ocys (BHR‐Ocys) and assessed the expression levels of Tnfsf11, Csf1, Spp1, and SOST at protein levels. The Western blotting results indicated that the expression of Tnfsf11, Csf1, Spp1, and SOST at the protein levels in BHR‐Ocys was significantly greater than in non‐BHR‐Ocys (**Figure** **3**A–E). These findings were consistent with the results from single‐cell sequencing analysis. Given that PMOP is characterized by the overactivity of osteoclasts, we co‐cultured BHR‐Ocys and non‐BHR‐Ocys with BMDMs (Figure 3F). TRAP staining results revealed that BHR‐Ocys promoted the differentiation of BMDMs into osteoclasts, whereas non‐BHR‐Ocys did not influence osteoclasts differentiation, indicating that BHR‐Ocys was a key subgroup regulating osteoclast activity among all osteocytes (Figure 3G,H).

We performed subgroup analysis of macrophages in the single‐cell dataset and identified the osteoclast population characterized by the genes Ctsk, Mmp9, Acp5, and Nfatc1 (Figure S7A–C, Supporting Information). To investigate the interactions between different osteocyte subclusters and osteoclasts in sham and OVX mice, we conducted additional analysis using CellChat. The results indicated significant alterations in both the strength and number of interactions between BHR‐Ocys and osteoclasts (Figure 3I–K).

**Figure 4:**
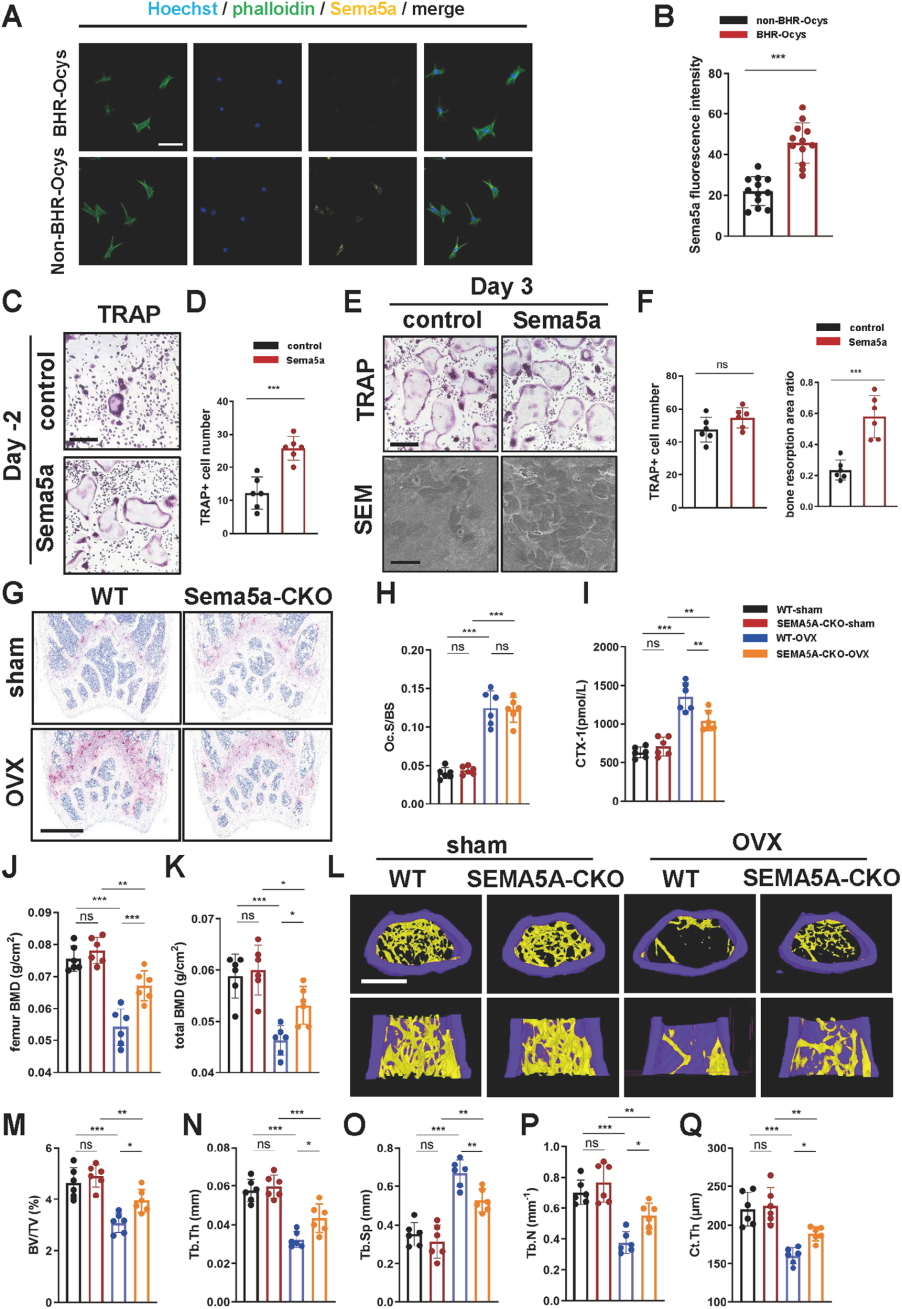
BHR‐Ocys promoted osteoclast activation through Sema5a, thereby leading to bone loss. A) Representative images of immunofluorescence staining of cultured BHR‐Ocys and non‐BHR‐Ocys osteocytes. B) Statistics of fluorescence intensity of Sema5a (three randomly selected viewing fields were evaluated per sample, *n* = 3 per group, ≈100 cells were counted per group; scale bar, 50 µm). C) Representative images of TRAP staining of BMDMs (scale bar, 200 µm). BMDMs were treated with 10 µg mL^−1^ Sema5a and 10 ng mL^−1^ M‐CSF for 2 days, and cells were further cultured with 10 ng mL^−1^ M‐CSF and a suboptimal dose of RANKL (5 ng mL^−1^) for 3 days. D) Quantification of TRAP‐positive multinucleated (≥3 nuclei) cell number per well (*n* = 3 per group). E) Representative images of TRAP staining of BMDMs (scale bar, 200 µm) and SEM of bone slices (scale bar, 50 µm). BMDMs were treated with 50 and 10 ng mL^−1^ M‐CSF for 3 days. Subsequently, 10 ng mL^−1^ Sema5a was added, and the cells are cultured for an additional two days. F) Quantification of TRAP‐positive multinucleated (≥3 nuclei) cell number per well (*n* = 6 per group) and ratio of bone resorption area (*n* = 6 per group). G) Representative images of TRAP staining in the distal femoral metaphysis of mice in each group (scale bar, 500 µm). H) The osteoclast surface/bone surface (Oc.S/BS) values in the distal femurs of mice in each group (*n* = 6 per group). I) Serum CTX‐1 levels of mice in each group (*n* = 6 per group). J) Femur BMD and K) total BMD of each group of mice were assessed using DXA (*n* = 6 per group). L) Representative μ‐CT images of distal femurs (scale bar, 500 µm). (M) Distal femur BV/TV, (N) Tb.Th, O) Tb.Sp, P) Tb.N, and Q) Ct.Th were measured by μ‐CT in mice in each group (*n* = 6 per group). Sema5a‐CKO: Dmp1^Cre^; Sema5a*^fl/fl^* mice. Data are represented as the mean ± SD. *p < 0.05, **p < 0.01, ***p < 0.001, ns = not significant. Statistical analysis employed two‐tailed unpaired Student's *t*‐test or ANOVA with post‐hoc Tukey–Kramer test.

Receptor‐ligand analysis suggested that the Sema5a‐Plxna1 axis may serve as a crucial signaling pathway for communication between BHR‐Ocys and osteoclasts in OVX mice (Figure S8, Supporting Information). To clarify the role of the Sema5a‐Plxna1 axis in maintaining bone homeostasis, BHR‐Ocys and other osteocytes (non‐BHR‐Ocys) isolated from OVX mice were cultured. The isolated osteocytes did not proliferate in culture, consistent with their terminal differentiation state. In addition, characteristic dendritic processes in cultured osteocytes were observed. The cultured cells were confirmed to be osteocytes. The results of immunofluorescence staining further confirmed that BHR‐Ocys significantly overexpressed Sema5a compared to non‐BHR‐Ocys (**Figure** **4**A,B). We pretreated BMDMs with recombinant Sema5a for two days, followed by induction with low concentrations of RANKL (5 ng/mL). After four days of induction, Sema5a‐pretreated BMDMs showed significant osteoclastogenesis compared to the untreated control group (Figure 4C,D). In contrast, introduction of Sema5a on the third day of osteoclast induction had no significant effect on osteoclast differentiation but still promoted the bone resorption capacity of osteoclasts (Figure 4E,F). Notably, induction of BMDMs with Sema5a alone did not suffice to trigger osteoclast differentiation, underscoring the necessity of RANKL for Sema5a to facilitate both the differentiation and resorption activity of osteoclasts (Figure S9, Supporting Information). To further elucidate the role of osteocyte‐derived Sema5a in osteoporosis, we generated osteocyte‐specific Sema5a knockout mice (Sema5a‐CKO). TRAP staining indicated that the activity of osteoclasts in Sema5a‐CKO mice did not significantly differ without OVX modeling. However, after OVX modeling, Sema5a‐CKO mice exhibited a significant decrease in Oc.S/BS compared to the WT group (Figure 4G,H). Serum CTX‐1 changes were consistent with the TRAP staining findings (Figure 4I). Dual‐energy X‐ray assessments revealed that after OVX modeling, femur BMD and total BMD in Sema5a‐CKO mice increased compared to WT littermates, indicating significant improvements in bone loss (Figure 4J,K). Lastly, we evaluated the bone microstructure of each mouse group using microCT and observed no substantial differences in bone microstructure parameters between WT and Sema5a‐CKO mice without OVX modeling. Following OVX modeling, Sema5a‐CKO mice demonstrated significant improvements in BV/TV, Tb.N, Tb.Th, and Ct.Th, while Tb.Sp significantly decreased (Figure 4L–Q). These findings suggested that the knockout of osteocyte‐derived Sema5a inhibited osteoclast activity, thereby improving bone microstructure and mitigating bone mass loss.

Given that osteocyte‐derived Sema5a may also influence osteoblasts, we treated MC3T3‐E1 osteoblasts with Sema5a. RT‐qPCR results indicated no statistically significant effects of Sema5a on osteogenic differentiation markers, including ALP, Runx2, OCN, and OPN (Figure S10A–D, Supporting Information). ALP staining further confirmed that Sema5a exerted no substantial impact on osteoblast differentiation (Figure S10E,F, Supporting Information). ARS staining results indicated that Sema5a did not significantly alter the mineralization capacity of osteoblasts (Figure S10G,H, Supporting Information). Consequently, osteocyte‐derived Sema5a primarily affected osteoclast function rather than osteoblast activity.

### 2.3 Plxna1 was Crucial for Sema5a‐Induced Osteoclastic Differentiation in BMDMs

**Figure 5:**
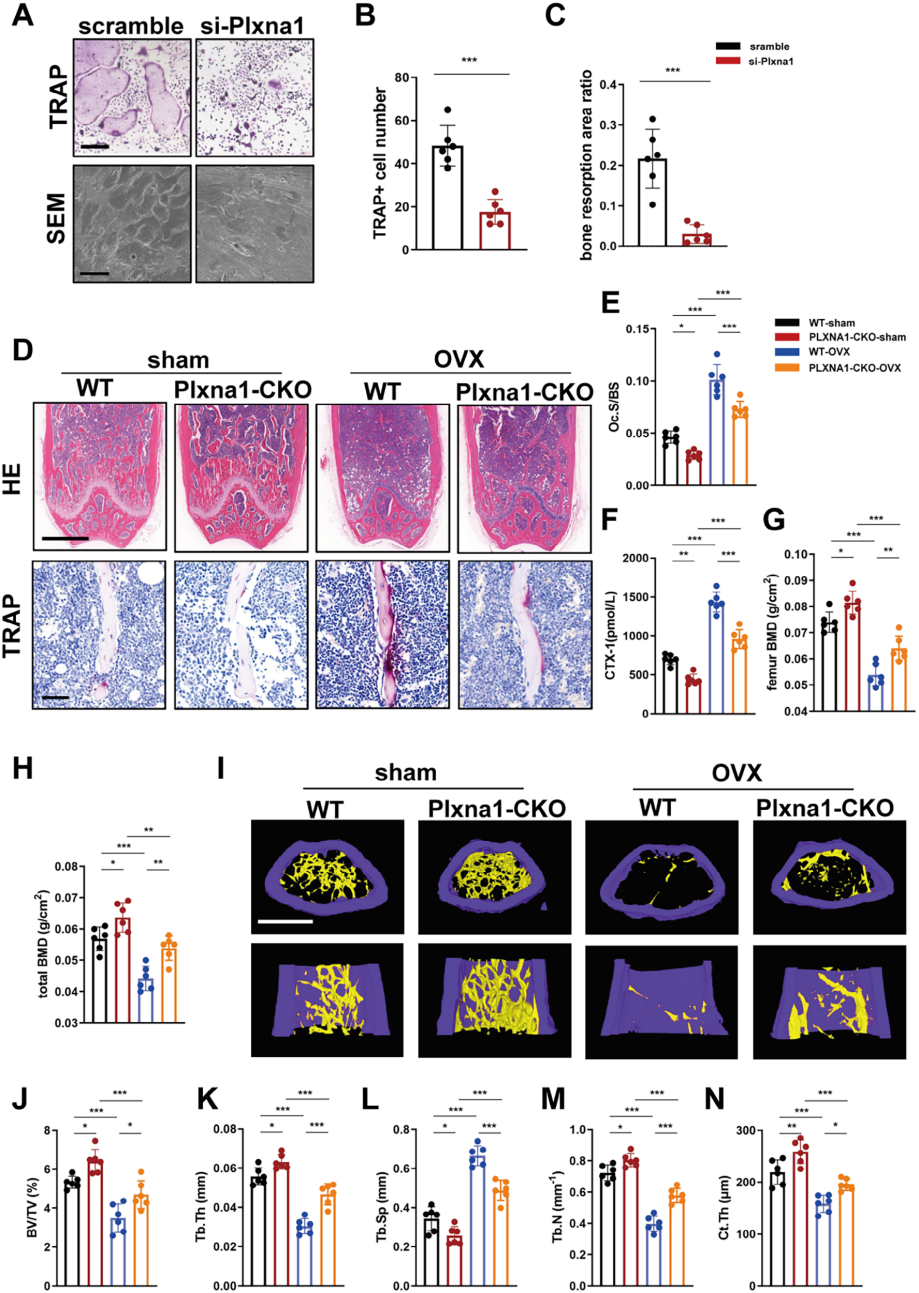
Plxna1 in BMDMs is crucial for osteoclast‐mediated bone resorption. A) Representative images of TRAP staining of BMDMs (scale bar, 200 µm) and SEM of bone slices (scale bar, 50 µm). BMDMs were transferred with si‐Plxna1 or scramble siRNA and cultured with 50 and 10 ng mL^−1^ M‐CSF for 5 days. B) Quantification of TRAP‐positive multinucleated (≥3 nuclei) cell number per well (*n* = 6 per group). C) Quantification of ratio of bone resorption area (*n* = 6 per group). D) Representative images of HE (scale bar, 500 µm) and TRAP (scale bar, 50 µm) staining in the distal femurs of mice in each group. E) The Oc.S/BS values in the distal femurs of mice in each group (*n* = 6 per group). F) Serum CTX‐1 levels of mice in each group (*n* = 6 per group). G) Femur BMD and H) total BMD of each group of mice were assessed using DXA (*n* = 6 per group). H) Representative μ‐CT images of distal femurs (scale bar, 500 µm). I) Distal femur BV/TV, J) Tb.Th, K) Tb.Sp, L) Tb.N, and M) Ct.Th were measured by μ‐CT in mice in each group (*n* = 6 per group). Plxna1‐CKO: Lyz2^Cre^; Plxna1*^fl/fl^* mice. Data are represented as the mean ± SD. *p < 0.05, **p < 0.01, ***p < 0.001. Statistical analysis employed two‐tailed unpaired Student's *t*‐test or ANOVA with post‐hoc Tukey–Kramer test.

The results of receptor‐ligand analysis suggested that Plxna1 in osteoclasts served as a crucial receptor protein for Sema5a derived from osteocytes. To elucidate this, we knocked down Plxna1 in BMDMs and found that Sema5a could not facilitate the differentiation and bone resorption of BMDMs with Plxna1 knocked down (**Figure** **5**A–C). We employed Lyz2‐Cre transgenic mice to specifically target Plxna1 deletion in osteoclasts (Plxna1‐CKO). TRAP staining results indicated that both OVX unmodeled and modeled conditions resulted in a significant decrease in the Oc.S/BS of Plxna1‐CKO mice (Figure 5D,E). Serum CTX‐1 levels changed in accordance with the TRAP staining results (Figure 5F). Dual‐energy X‐ray analysis demonstrated that, compared to WT littermates, Plxna1‐CKO mice exhibited increased femoral bone density and total bone density, indicating a notable improvement in bone loss following the deletion of Plxna1 in osteoclasts (Figure 5G,H). Finally, we assessed the bone microstructure of each group of mice using microCT. Both OVX unmodeled and modeled conditions showed significant improvements in BV/TV, Tb.N, Tb.Th, and Ct.Th of Plxna1‐CKO mice, while Tb.Sp significantly decreased (Figure 5I–N). These results underscore the importance of Plxna1 in the differentiation of osteoclasts.

**Figure 6:**
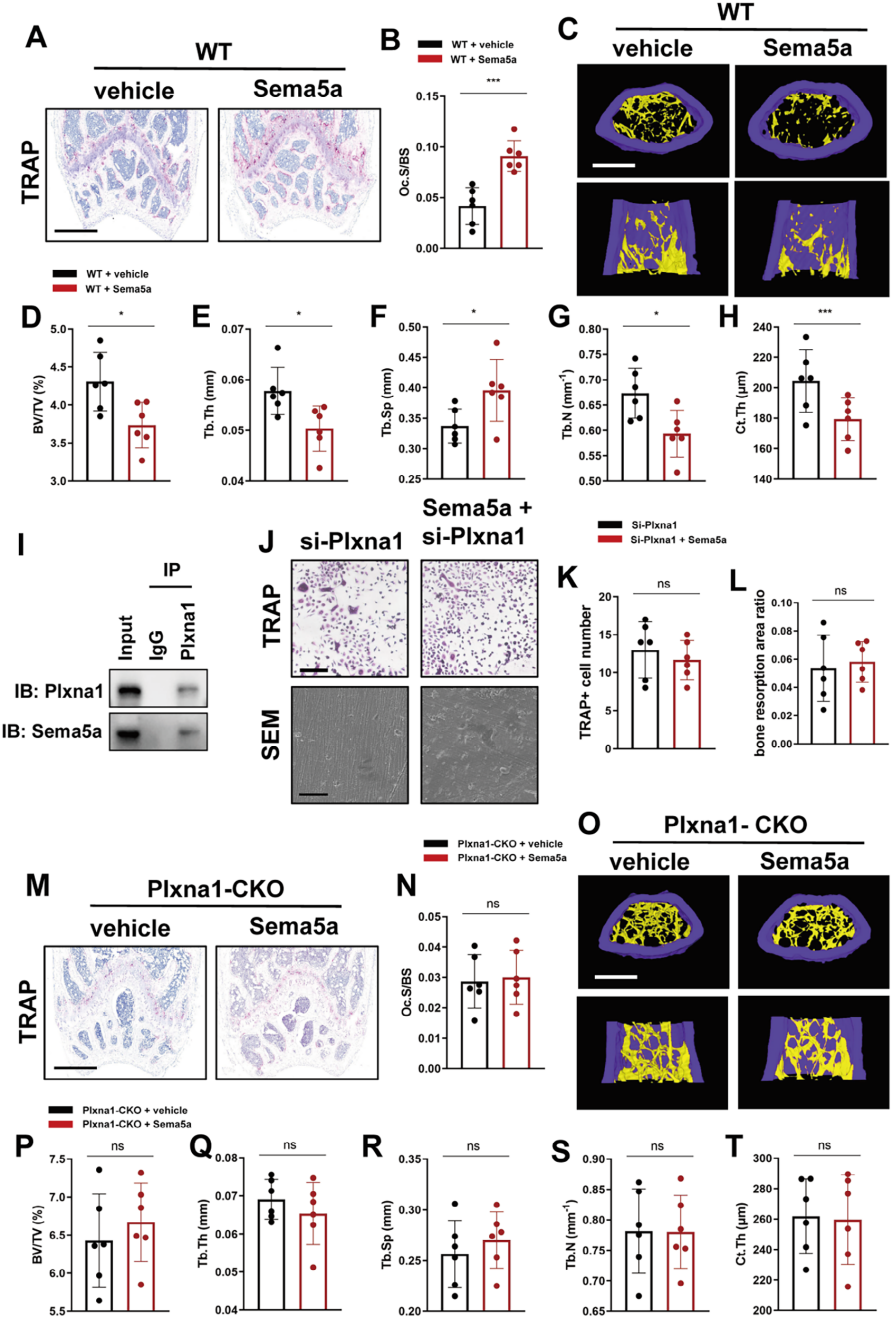
Sema5a promotes osteoclast differentiation and bone resorption by binding to its receptor Plxna1. A) Representative images of TRAP staining in the distal femurs of WT mice treated with or without Sema5a (scale bar, 500 µm). Sema5a injection was performed intraperitoneally at a dose of 100 ng g^−1^ body weight, every 3 days, for a total of two months. (scale bar, 200 µm). B) The Oc.S/BS values in the distal femurs of WT mice treated with or without Sema5a (*n* = 6 per group). C) Representative μ‐CT images of distal femurs of WT mice treated with or without Sema5a (scale bar, 500 µm). D) Distal femur BV/TV, E) Tb.Th, F) Tb.Sp, G) Tb.N, and H) Ct.Th were measured by μ‐CT in mice treated with or without Sema5a (*n* = 6 per group). I) Western blot analysis of co‐immunoprecipitation (co‐IP) assay result confirming the binding of Sema5a‐Plxna1 in BMDMs. J) Representative images of TRAP staining of BMDMs (scale bar, 200 µm) and SEM of bone slices (scale bar, 50 µm). BMDMs transferred with si‐Plxna1 were treated with or without Sema5a, and cultured with 50 and 10 ng mL^−1^ M‐CSF for 5 days. K) Quantification of TRAP‐positive multinucleated (≥3 nuclei) cell number per well (*n* = 6 per group). L) Quantification of ratio of bone resorption area (*n* = 6 per group). M) Representative images of TRAP staining in the distal femurs of Plxna1‐CKO mice treated with or without Sema5a (scale bar, 500 µm). Sema5a injection was performed intraperitoneally at a dose of 100 ng g^−1^ body weight, every 3 days, for a total of two months. (scale bar, 200 µm). (N) The Oc.S/BS values in the distal femurs of WT littermates treated with or without Sema5a (*n* = 6 per group). (O) Representative μ‐CT images of distal femurs of WT littermates treated with or without Sema5a (scale bar, 500 µm). P) Distal femur BV/TV, Q) Tb.Th, R) Tb.Sp, S) Tb.N, and T) Ct.Th were measured by μ‐CT in mice treated with or without Sema5a (*n* = 6 per group). Plxna1‐CKO: Lyz2^Cre^; Plxna1*^fl/fl^* mice. Data are represented as the mean ± SD. *p < 0.05, **p < 0.01, ***p < 0.001. ns = not significant. Statistical analysis employed two‐tailed unpaired Student's *t*‐test.

TRAP staining revealed that Sema5a could induce activation of osteoclasts in WT mice (**Figure** **6**A,B). Additionally, microCT results indicated that BV/TV, Tb.N, Tb.Th, and Ct.Th significantly decreased, while Tb.Sp significantly increased in Sema5a‐treated WT mice (Figure 6C–H). The results above suggested that Sema5a could lead to osteoclast activation and bone loss in vivo. The results of co‐immunoprecipitation experiment confirmed the binding of Sema5a to Plxna1 in BMDMs, suggesting that Sema5a may exert regulatory effects through its interaction with Plxna1(Figure 6I). However, when we treated Plxna1‐knockdown BMDMs with Sema5a, the ability of Sema5a to promote osteoclast differentiation and bone resorption was abolished (Figure 6J–L). In addition, TRAP staining did not reveal evidence of osteoclast activation after treating Plxna1‐deficient mice with Sema5a (Figure 6M,N). Furthermore, microCT results indicated that Sema5a treatment did not significantly adversely affect the bone microstructure of Plxna1‐CKO mice (Figure 6O–T). These findings demonstrated that Sema5a promotes osteoclast differentiation and bone resorption through its interaction with the receptor Plxna1.

### 2.4 Sema5a‐Plxna1 Axis Promoted Osteoclast Differentiation by Activating the PI3K/AKT/Myc Pathway

**Figure 7:**
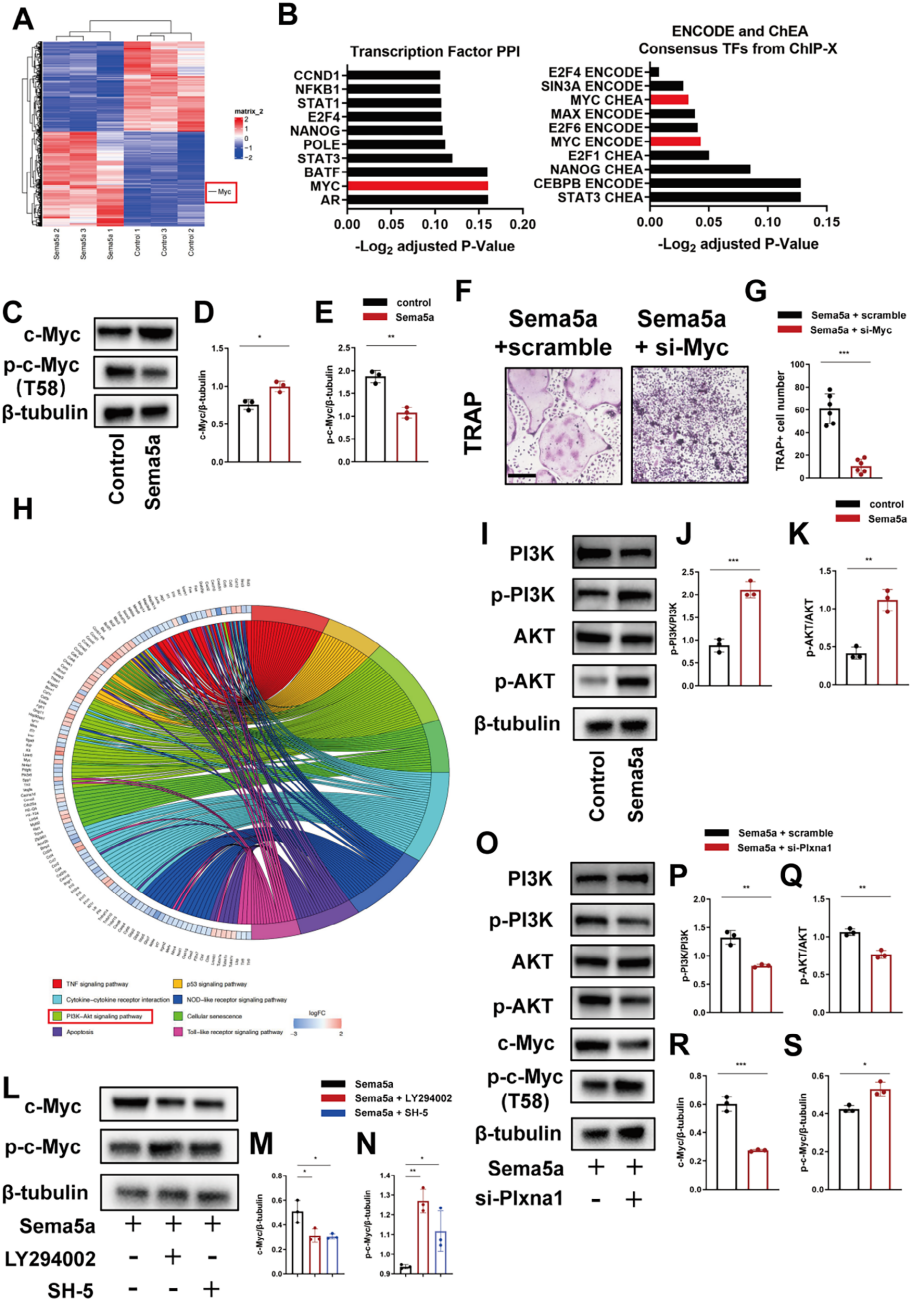
Sema5a‐Plxna1 upregulated Myc by activating the PI3K/AKT signaling pathway, thereby promoting osteoclast differentiation and bone resorption. A) The heatmap displayed DEGs in BMDMs treated with Sema5a. Myc was significantly upregulated in BMDMs after Sema5a treatment. B) DEGs were subjected to Enrichr analysis using Enrichr library (Transcription Factor PPI, ENCODE and ChEA Consensus TFs from ChIP‐X). Enriched transcription factors were shown. C) Representative Western blot and quantification of the protein level of D) Myc and E) phosphorylated Myc in BMDMs treated with or without Sema5a (*n* = 3 per group). F) Representative images of TRAP staining of BMDMs (scale bar, 200 µm). BMDMs transferred with si‐Myc were treated with or without Sema5a, and cultured with 50 and 10 ng mL^−1^ M‐CSF for 5 days. G) Quantification of TRAP‐positive multinucleated (≥3 nuclei) cell number per well (*n* = 6 per group). H) KEGG enrichment of DEGs showed the activation of PI3K/AKT signaling pathway was associated with the overexpression of Myc. I) Representative Western blot and quantification of the protein level of J) phosphorylated PI3K and K) phosphorylated AKT in BMDMs treated with or without Sema5a (*n* = 3 per group). M) Representative Western blot and quantification of the protein level of L) Myc and N) phosphorylated Myc in BMDMs treated with PI3K inhibitor (LY294002) or AKT inhibitor (SH‐5) (*n* = 3 per group). O) Representative Western blot and quantification of the protein level of P) phosphorylated PI3K, Q) phosphorylated AKT, R) Myc and S) phosphorylated Myc in BMDMs treated with Sema5a and transferred with or without si‐Plxna1 (*n* = 3 per group). Data are represented as the mean ± SD. *p < 0.05, **p < 0.01, ***p < 0.001. ns = not significant. Statistical analysis employed two‐tailed unpaired Student's *t*‐test or ANOVA with post‐hoc Tukey–Kramer test.

To investigate the specific molecular mechanisms by which Sema5a facilitated osteoclast differentiation, we conducted transcriptome sequencing following the treatment of BMDMs with Sema5a. The heat map displaying differentially expressed genes was illustrated in **Figure** **7**A. Myc exhibited significant upregulation in BMDMs treated with Sema5a.

We then performed Enrichr analysis on the differentially expressed genes (DEGs) and identified Myc as one of the most upregulated transcription factors in Sema5a‐treated BMDMs (Figure 7B). Western blot analysis demonstrated that Myc was upregulated while the phosphorylation level at the T58 site of c‐Myc was significantly reduced in BMDMs exposed to Sema5a (Figure 7C–E). Knockdown of Myc inhibitors effectively attenuated the stimulatory effect of Sema5a on osteoclast differentiation (Figure 7F,G). KEGG enrichment analysis of DEGs was performed to investigate the specific molecular mechanisms by which the Sema5a‐Plxna1 axis regulates c‐Myc phosphorylation. The results revealed significant enrichment of the PI3K/AKT signaling pathway (Figure 7H). Furthermore, Western blot results confirmed that Sema5a treatment activated the PI3K/AKT signaling pathway in BMDMs (Figure 7I–K). Following intervention with PI3K and AKT inhibitors, Myc protein level was significantly reduced and Myc phosphorylation level at T58 site was significantly increased in Sema5a‐treated BMDMs, demonstrating that inhibition of the PI3K/AKT pathway effectively counteracts the regulatory influence of Sema5a on Myc (Figure 7L–N). Knockdown of Plxna1 in BMDMs diminished the capacity of Sema5a to regulate Myc expression and phosphorylation, indicating that Sema5a requires interaction with Plxna1 for the regulation of Myc (Figure 7O–S). Schematic illustration of the PI3K/AKT/c‐Myc signaling pathway activated by Sema5a was shown in Figure S11 (Supporting Information). Overall, these findings suggested that Sema5a promoted osteoclast differentiation by binding to Plxna1 and thus activating the PI3K/AKT pathway to enhance Myc‐mediated osteoclast differentiation.

### 2.5 Targeting the Sema5a‐Plxna1 Axis Effectively Alleviated Estrogen Deficiency‐Induced Bone Loss

The ovariectomized (OVX) model utilized in this study is primarily designed to simulate the progression of postmenopausal osteoporosis (PMOP) resulting from estrogen deficiency. We hypothesize that the upregulation of the BHR‐Ocys ratio is closely associated with decreased estrogen levels. The Esr1 expression level in BHR‐Ocys was significantly higher than in other osteocytes, suggesting a strong correlation between BHR‐Ocys and estrogen. We thus hypothesized that the upregulation of the BHR‐Ocys ratio is closely associated with estrogen deficiency.

**Figure 8:**
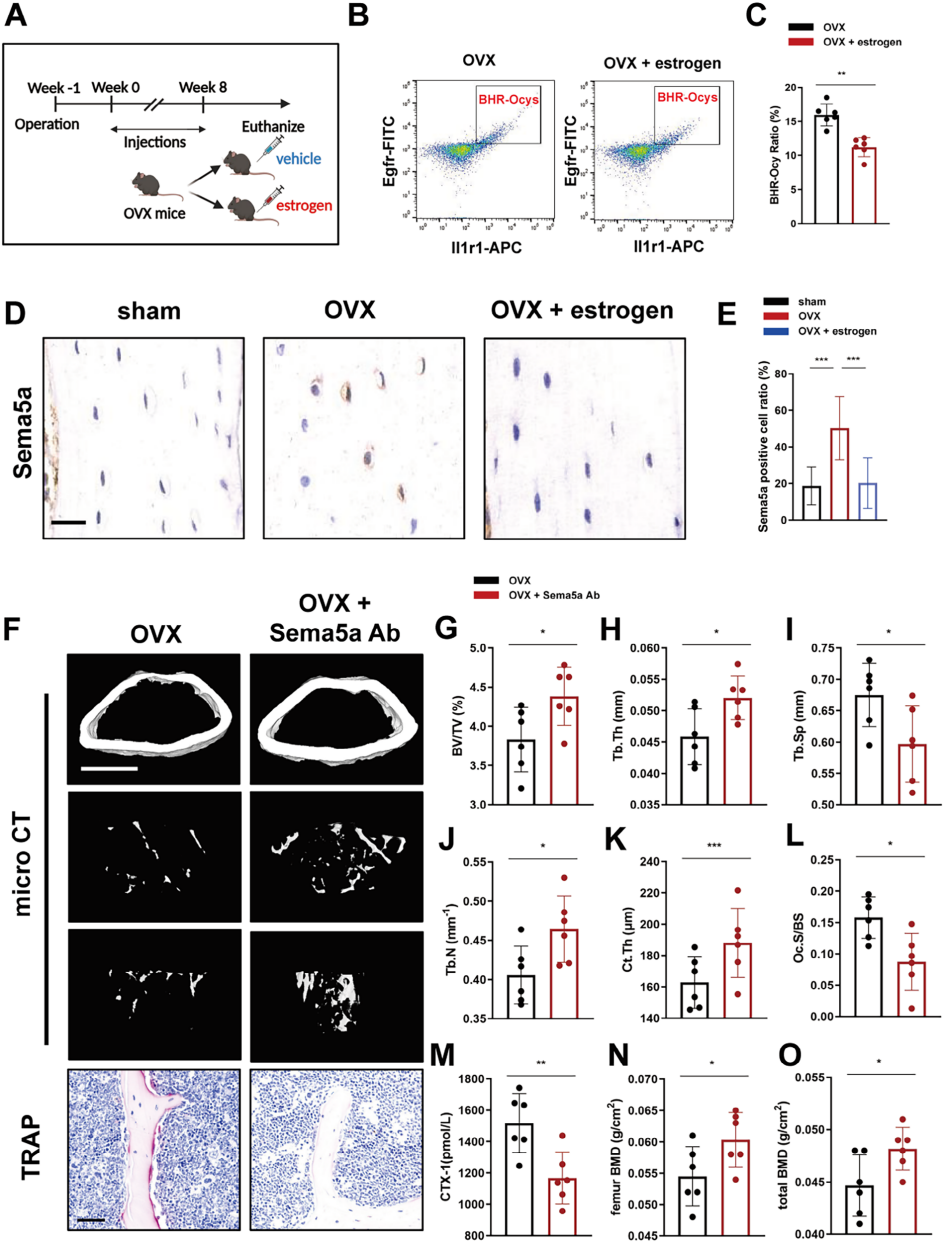
Targeting the Sema5a‐Plxna1 axis ameliorates bone loss induced by estrogen deficiency. A) Schematic showing the experimental protocol for 8 weeks of estrogen (17β‐estradiol) treatment in mice. B) Flow cytometry sorting of BHR‐Ocys from femurs of OVX mice treated with or without estrogen. C) Statistical analysis showing the ratios of BHR‐Ocys osteocytes in OVX mice treated with or without estrogen (*n* = 6 per group). D) Representative images of IHC staining for detecting Sema5a in osteocytes in the femurs of OVX mice treated with or without estrogen (scale bar, 20 µm). E) Quantification of Sema5a‐positive osteocytes (five randomly selected viewing fields were evaluated per sample, *n* = 6 per group). F) Representative images of TRAP staining (scale bar, 50 µm) and μ‐CT images (scale bar, 500 µm) of distal femurs of OVX mice treated with Sema5a antibody or Myc inhibitor (JQ1). G) Distal femur BV/TV, H) Tb.Th, I) Tb.Sp, J) Tb.N, and K) Ct.Th were measured by μ‐CT (*n* = 6 per group). L) Statistics of the Oc.S/BS values (*n* = 6 per group). M) Statistics of the Oc.S/BS values (*n* = 6 per group). M) Statistics of the serum CTX‐1 level (*n* = 6 per group). N) Statistics of the femur BMD (*n* = 6 per group). O) Statistics of the total BMD (*n* = 6 per group). Data are represented as the mean ± SD. *p < 0.05, **p < 0.01, ***p < 0.001. Statistical analysis employed two‐tailed unpaired Student's *t*‐test or ANOVA with post‐hoc Tukey–Kramer test.

To investigate the effects of estrogen on BHR‐Ocys, we administered 17β‐estradiol to OVX mice. The schematic diagram of the in vivo estradiol rescue experiment is illustrated in **Figure** **8**A. Flow cytometry analyses revealed that estradiol supplementation effectively reduces the BHR‐Ocys ratio (Figure 8B,C). Additionally, IHC results demonstrated that estradiol supplementation significantly lowers the expression of osteocyte Sema5a (Figure 8D,E). Moreover, the levels of Sema5a in bone tissues of PMOP patients were significantly higher than those in the control group (the data were obtained from GSE230665, Figure S12, Supporting Information). Finally, intervening with Sema5a antibodies effectively prevented the decline in BV/TV, Tb.N, Tb.Th, and Ct.Th as well as the increase in Tb.Sp, thereby inhibiting osteoclast‐mediated bone resorption and mitigating the deterioration of bone microstructure in OVX mice (Figure 8F–M). Furthermore, the Sema5a antibody significantly suppressed bone loss in OVX mice (Figure 8N,O), indicating that targeting the Sema5a‐Plxna1 axis may offer an effective strategy for PMOP.

## 3 Discussion

Cell subpopulations refer to distinct groups of cells within a larger population that exhibit unique characteristics, behaviors, or functions, which is a phenomenon observed across various organisms and tissues. The concept of cell subpopulations is pivotal in understanding the complexity and functionality of various biological systems. This heterogeneity is crucial for the adaptability and resilience of biological systems, allowing them to respond to environmental changes and challenges.^[^ ^14^, ^15^ ^]^ Investigating the characteristics and variations of cell subpopulations under different physiological and pathological conditions can deepen our understanding of intercellular interactions and their potential effects on disease progression. In osteoporosis‐related study, the diversity and functional attributes of bone cell subpopulations have gained increasing attention.^[^ ^16^, ^17^ ^]^ Bone cells from different regions of bone tissue may perform distinct roles in bone metabolism. For instance, skeletal stem cells represent not a single cell type but rather a related family of cells with varying anatomical locations and functions.^[^ ^18^ ^]^ Recent studies have also revealed functional differences among osteocytes within bone. The lacunar networks of osteocytes across various regions of the femoral neck exhibit significant variability, which may influence bone quality and fragility.^[^ ^19^ ^]^ The response of osteocytes to hypoxic stress varies depending on their location in the cortex, with osteocytes located near the bone membrane and endosteum demonstrating higher mitochondrial content and activity compared to mid‐cortical osteocytes.^[^ ^20^ ^]^ Cortical osteocytes primarily utilize aerobic (mitochondrial) pathways to maintain normal oxidative states, indicating that the oxidative functions of osteocytes exhibit regional differences. This suggests that osteocytes with distinct functions may play unique roles in bone metabolism and the onset of osteoporosis. However, research on osteocyte subpopulations is still limited, and the significance of osteocyte heterogeneity in osteoporosis remains poorly understood.

Traditionally, osteocytes have been viewed as a collective group of cells that sense mechanical stress to maintain bone metabolism and mineral balance. However, our research uncovered the existence of a novel specialized osteocyte subpopulation primarily responsible for regulating bone homeostasis. This subpopulation significantly expressed classical regulators of osteoblasts and osteoclasts, including RANKL, SPP1, CSF1, and SOST, which served crucial roles in bone formation and resorption.^[^ ^5^, ^21^, ^22^, ^23^, ^24^, ^25^ ^]^ An additional observation worth of mention was the very low level of OPG expression in BHR‐Ocys, which may further explain the potent osteoclastogenesis (data not shown). Furthermore, there were specialized subpopulations responsible for matrix synthesis and stress sensing, suggesting that the functions of osteocytes were not entirely uniform and were likely related to their anatomical positions and microenvironments. Based on surface markers, we designated this osteocyte subpopulation (Egfr+ Il1r1+ Sema5a+ osteocyte) as BHR‐Ocys. Interestingly, we observed that the BHR‐Ocys tended to distribute closer to the bone surface rather than in deeper regions. This positional preference may facilitate their secretion of bone homeostasis‐regulating cytokines, such as Sema5a as we proposed, toward the bone surface. The characteristics and underlying reasons for this distribution pattern require further investigation. When co‐cultured with BMDMs, the BHR‐Ocys from OVX mice showed the ability to promote osteoclast differentiation, while non‐BHR‐Ocys did not, suggesting the BHR‐Ocys was the key osteocyte subpopulation that caused bone loss in the pathological process of osteoporosis. Notably, the proportion of BHR‐Ocys significantly increased in the femurs of postmenopausal osteoporotic mice, further indicating its crucial role in the onset and progression of osteoporosis. ESR1 is a critical receptor for estrogen. Our data demonstrated that BHR‐Ocys exhibited high ESR1 expression, which may partially explain why this subset was activated under estrogen deficiency. Furthermore, given the elevated IL1R1 expression in BHR‐Ocys, chronic inflammation induced by estrogen deficiency could represent an additional factor contributing to BHR‐Ocys activation.^[^ ^26^ ^]^ However, since the role of EGFR in PMOP remains not well characterized, whether the high Egfr expression in BHR‐Ocys correlates with their activation in PMOP requires further investigation.

To investigate the cell‐to‐cell interaction pathways between various osteocyte subsets and other cell types in the bone marrow, we applied CellChat analysis to evaluate the differential *number* of interactions and their strength in sham and OVX mice. Pathways with the most significant changes in interaction strength often represent core mechanisms driving phenotypic changes, such as enhanced bone resorption. Therefore, compared to the differential number of interactions, differential interaction strength can be used to identify key regulatory nodes. Our CellChat revealed that among all osteocyte subpopulations, the interaction strength between BHR‐Ocys and osteoclasts exhibited the most pronounced alterations, suggesting that BHR‐Ocys may play a non‐negligible role in the formation and activation of osteoclasts in PMOP. Based on the Cellchat results, we further explored novel mechanisms by which osteocytes participate in regulating bone homeostasis. The semaphorin family comprises important cell signaling molecules, initially discovered as axon guidance factors during nervous system development. However, recent research has highlighted their critical roles in skeletal development and bone metabolism. Semaphorins regulate the balance between bone formation and resorption by binding to their receptors (such as neuronal receptors and plexins), thereby influencing bone density and quality.^[^ ^27^ ^]^ Semaphorin 3a (Sema3a) not only inhibits osteoclast formation but also promotes osteoblast differentiation, thereby playing dual roles in bone remodeling.^[^ ^28^, ^29^ ^]^ Semaphorin 4d (Sema4d), through binding to Plexin‐B1, is significantly involved in both bone resorption and regeneration, and inhibiting its signaling pathways could improve the pathological status of diseases like osteoarthritis.^[^ ^30^, ^31^ ^]^ Semaphorin 5a is one of the prominent members of the semaphorin family, with recent studies indicating its significance in cancer and autoimmune diseases.^[^ ^32^, ^33^ ^]^ However, its role in bone metabolism remains unclear. We found that Sema5a was highly expressed in BHR‐Ocys, which may have signified the importance of Sema5a in bone metabolism. Previous reports suggest that Sema5a may influence signal transduction networks by interacting with various cell surface receptors, impacting cell growth, differentiation, and function. CellChat analysis also suggests that the Sema5a‐Plxna1 axis might serve as an important signaling pathway for communication between BHR‐Ocys and osteoclasts in the current study. Consequently, we first investigated the role of Sema5a derived from osteocytes in bone remodeling. We discovered that Sema5a alone does not induce BMDMs to differentiate into osteoclasts but enhances BMDM differentiation into osteoclasts in the presence of RANKL. Furthermore, osteoclasts treated with Sema5a displayed an increased capacity for bone resorption. Compared with the control group of ovariectomized mice, the ovariectomized mice with osteocyte‐specific knockout of Sema5a exhibited decreased bone resorptive activity, increased bone mineral density, and improved bone microstructure, indicating that Sema5a derived from osteocytes is a critical factor promoting bone resorption in ovariectomized mice.

Sema5a dynamically regulates developmental and pathological processes through both membrane‐bound and soluble forms, a mechanism analogous to RANKL. Studies indicated that osteocytes serve as the primary cellular source of RANKL. Notably, membrane‐bound RANKL (mRANKL), rather than its soluble counterpart, predominantly mediates osteoclast regulation.^[^ ^34^, ^35^ ^]^ Osteocytes establish efficient intercellular communication via their specialized dendritic processes, a mechanism that plays pivotal roles in skeletal homeostasis and repair. These cellular projections enable direct contact with bone‐surface‐residing cells, providing the structural basis for membrane‐bound proteins (including mRANKL) to exert regulatory functions on target cells.^[^ ^4^ ^]^ Although our current study cannot definitively determine whether osteocyte‐derived Sema5a exists in membrane‐bound or secreted form, our data provide compelling evidence that osteocyte‐derived Sema5a functions as a critical regulator of osteoclastogenesis in PMOP. The relative contributions of membrane‐bound versus secreted Sema5a, or potential synergistic effects of both isoforms, warrant further investigation in subsequent studies.

We further investigated the mechanisms by which Sema5a promotes osteoclast differentiation. Based on the results of CellChat analysis, we hypothesized that Plxna1 serves as the receptor for Sema5a derived from BHR‐Ocys. Our findings indicated that knocking down Plxna1 in BMDMs inhibited osteoclast differentiation. Studies have also shown that Plxna1 knockout mice exhibit increased bone mass phenotypes.^[^ ^36^ ^]^ Utilizing Lyz2‐Cre transgenic mice, we specifically deleted Plxna1 in osteoclasts. The results confirmed that the specific knockout of Plxna1 inhibited osteoclast differentiation and bone resorption, thereby improving bone mass in mice. This finding suggests that Plxna1 plays a vital role in regulating osteoclast differentiation. Notably, the positive effect of Sema5a on osteoclasts was significantly reduced following the knockout of Plxna1, indicating that Sema5a primarily exerts its regulatory effect on osteoclasts through its interaction with Plxna1. Although Sema5a‐treated osteoblasts exhibited changes in the expression levels of osteoblast‐related genes, these changes were insufficient to promote osteoblast differentiation and mineralization, as confirmed by the specific knockout of Sema5a in osteocytes. Prior research has also shown that deleting Plxna1 in mice does not influence bone formation,^[^ ^36^ ^]^ suggesting that Sema5a derived from osteocytes principally regulates osteoclast‐mediated bone resorption rather than osteoblast‐mediated bone formation.

Our research results on the molecular mechanism indicated that the upregulation of Myc was the key point for the Sema5a‐plxna1 axis to promote osteoclast differentiation. Myc is an important proto‐oncogene that plays a key role in a variety of cellular activities, including cell cycle regulation, apoptosis, DNA damage response, and hematopoiesis.^[^ ^37^, ^38^ ^]^ Knocking down Myc can significantly inhibit the osteoclast differentiation‐promoting effect of Sema5a, indicating that Myc plays an important role in the formation of osteoclasts mediated by the Sema5a‐Plxna1 axis. Studies have shown that Myc plays an important regulatory role in the formation and function of osteoclasts. Deleting Myc in osteoclasts increases bone mass and protects mice from ovariectomy‐induced osteoporosis.^[^ ^39^ ^]^ During osteoclast formation, Myc can influence osteoclast differentiation by regulating specific signaling pathways. For example, Myc promotes RANKL‐induced osteoclast formation by regulating the miR‐320a/PTEN pathway.^[^ ^40^ ^]^ Myc can also regulate the transcriptional program of osteoclast development by cooperating with other transcription factors such as Brd2/4 and Max, thereby affecting osteoclast activation.^[^ ^41^ ^]^ In addition, Myc is elevated in rheumatoid arthritis macrophages, which is important for osteoclastogenesis and TNF‐induced bone resorption.^[^ ^42^ ^]^ The transcription, translation, post‐translational modifications, stability, and degradation of Myc are regulated sequentially by the RAS‐MAPK pathway and the PI3K/AKT pathway.^[^ ^43^ ^]^ The newly synthesized Myc protein exhibits inherent instability, characterized by a short half‐life. Activated ERK enhances the stability of Myc by increasing phosphorylation at the S62 site.^[^ ^44^ ^]^ The activation of the PI3K/AKT signaling pathway reduces phosphorylation of Myc at the T58 site by inhibiting GSK3β activity, thereby decreasing Myc's degradation rate and enhancing its stability.^[^ ^45^, ^46^ ^]^ Our findings indicated that Sema5a promoted Myc‐mediated osteoclast differentiation by upregulating Myc transcription and activating the PI3K/AKT signaling pathway to reduce Myc phosphorylation at the T58 site, thus inhibiting its degradation.

The regulatory role of BHR‐Ocys in bone homeostasis presented a novel mechanism for maintaining the dynamic balance between osteoblasts and osteoclasts. Through intercellular signaling, BHR‐Ocys could effectively regulate osteoclast activity, preventing excessive resorption during peak periods and maintaining bone tissue health. The discovery of BHR‐Ocys provided new targets for future research and treatment of bone metabolism‐related diseases, with significant implications for osteoporosis prevention and treatment. Targeting Sema5a or its downstream signaling pathways could represent a promising direction for developing next‐generation anti‐osteoporosis medications. For instance, employing antibodies or small molecule inhibitors to target Sema5a may mitigate its stimulatory effects on osteoclasts, thereby reducing bone resorption rates and enhancing bone density. Additionally, interventions aimed at Plxna1 could effectively regulate osteoclast function, opening new avenues for osteoporosis treatment. In this study, we utilized a PMOP model and confirmed that the activation of BHR‐Ocys is associated with estrogen deficiency. Currently, there is insufficient evidence to determine whether BHR‐Ocys play a role in other bone diseases. Considering that in our study, some experiments which did not introduce estrogen as an intervention condition also indicated that the Sema5a‐Plxna1 axis significantly regulates osteoclast differentiation, we propose its potential involvement in other bone diseases related to bone homeostasis imbalance. This confers a particular value on the present research and warrants further investigation.

While this study proposed a relationship between the BHR‐Ocys and osteoporosis, there were limitations to this study that should be acknowledged. First, the specific molecular mechanisms through which Sema5a regulates osteoclast differentiation necessitate further exploration. In addition, Sema5a expression is significantly elevated in bone tissue from postmenopausal osteoporosis patients compared to individuals with normal bone mass, providing clinical evidence for the relevance of Sema5a in osteoporosis pathogenesis. However, further clinical studies are still needed to fully elucidate this relationship. Furthermore, future research should investigate functional heterogeneity within the BHR‐Ocys in different pathological states, which may elucidate whether targeting Sema5a has broader application potential beyond postmenopausal osteoporosis patients to include elderly individuals and other high‐risk groups.

In conclusion, this study provided a new perspective on the role of the osteocyte subpopulation in bone homeostasis and osteoporosis, suggesting that osteocytes have a more complex role in regulating bone metabolism and maintaining bone health. Further exploration reveals that the Sema5a‐Plxna1 axis served as a pivotal signaling pathway for BHR‐Ocys in regulating osteoclast differentiation and function, potentially offering new targets for therapeutic interventions in osteoporosis and laying the groundwork for future drug development.

## 4 Experimental Section

### Animal Model

Mice were adaptively fed for 1 week before the establishment of the ovariectomized (OVX) mouse model. Eight‐week‐old female mice were anesthetized by isoflurane inhalation. After successful anesthesia, the mouse was placed in a sterile operating area, and an incision was made in the back to expose the ovaries. The ovaries were carefully identified, and a bilateral ovariectomy was performed by excising both ovarian tissues. Incisions were closed with surgical sutures. The mice were closely monitored for recovery, and any signs of discomfort or complications were addressed promptly.

### Single‐Cell RNA Sequencing

Single‐cell RNA sequencing was performed by OEBiotech (Shanghai, China). After femoral separation in mice, digestion was performed to obtain single‐cell suspensions as reported previously.^[^ ^7^, ^47^, ^48^ ^]^ Briefly, the femurs from 6 mice (3 in sham group and 3 in OVX group) were harvested, and the soft tissues were carefully removed. Both ends of the femurs were cut, and the bone marrow cavity was repeatedly flushed with PBS to collect and preserve the bone marrow cells. To extract cells deeply embedded within the bone matrix, 1 mg mL^−1^ Collagenase I was dissolved in a‐MEM and 5 mM ethylenediaminetetraacetic acid (EDTA, Sigma) was dissolved in Ca^2+^‐ and Mg^2+^‐free Dulbecco's phosphate buffered solution containing 0.1% bovine serum albumin (BSA, Sigma,) with pH adjusted to 7.4. The femoral shafts were cut into ≈1 mm bone fragments. The bone fragments were then sequentially digested with collagenase I and EDTA solutions to release the cells. This sequential digestion process with EDTA and collagenase I was repeated three times following three rounds of collagenase I digestion. After each digestion step, the cells were collected by centrifugation. All harvested cells were treated with red blood cell lysis buffer. Freshly prepared cell suspensions were processed immediately in accordance with the manufacturer's instructions for the 10X Chromium 3′ v3 kit (10x Genomics, Pleasanton, CA). Library preparation and sequencing were conducted on the NovaSeq 6000 platform (Illumina, Inc., San Diego, CA) at GENERGY BIO (Shanghai, China).

### Read Alignment and Quality Control

Raw reads obtained from scRNA‐seq experiments were aligned to the mouse genome (mm10) using the CellRanger pipeline (cellranger‐7.0.1, 10X Genomics). To obtain a high‐quality data, cells with detected genes less than 500 and more than 7500 were removed for further analysis. In addition, cells with %Mitochondrial genes greater than 10% were removed to rule out apoptotic cells. R package “DoubletFinder” (version 2.0.3) was applied to remove potential doublets. Finally, there were 23491 genes and 51690 cells left for downstream analysis.

### Dimension Reduction and Clustering Analysis

After quality control, the preprocessed gene expression data of 51690 cells were analyzed by the Seurat package (version 4.2.0) and the following steps were performed in order: data normalization and transformation, highly variable gene selection, principal component analysis (PCA) and clustering. The count matrix was first normalized and transformed using function SCTransform. The top 3000 highly variable genes were then obtained by FindVariableGenes with the default variance stabilizing process. Top 30 principal components were used for clustering and the resolution of FindClusters was set to 0.4. To eliminate the batch effect, we performed harmony algorithm in Harmony R package before clustering analysis. Uniform Manifold Approximation and Projection (UMAP) was used for the final dimension reduction and visualization.

Marker genes were identified by comparing the mean expression of each gene in one cell type against mean of average expression in all other cell types by using function FindAllMarkers with the parameter method = MAST in Seurat package. Top marker genes were selected according to the adjusted p‐value and log2 fold change (log2 FC) within each cluster and cell clusters were determined based on previously known cell type marker expression.

### Differential‐Expression Analysis

Within each cluster, we detected DEGs between sham and OVX conditions by using “FindMarkers” function with parameter “test.use = MAST”. Then we set threshold *p* < 0.05, Fold change >= 1.2 to filter DEGs and obtained OVX up‐ and down‐regulated genes compared to Sham for each cluster. The DEGs functional enrichment analysis based on GO and KEGG was applied by an R package ClusterProfile (version 4.6.1) using a hypergeometric test and corrected for multiple hypothesis by FDR.

### Pseudotime Trajectory Analysis

Pseudotime trajectory inference of osteoblast and osteocyte population was carried out using the workflow suggested in the Monocle2 tutorial. In brief, the newCellDataSet function was applied to generate a data structure for trajectory analysis. Then, after normalization, reduceDimension and orderCells were used to reduce the dimensionality and order cells in the pseudotime trajectory. The genes used for pseudotime trajectory analysis came from the differentialGeneTest function (FDR adjusted *p*‐value < 0.01).

### Cell–Cell Communication Analysis

CellChat analysis was conducted to determine the intercellular communication changes between Sham and OVX. The R CellChat package (version 1.6.1) was used according to tutorials provided on GitHub (https://htmlpreview.github.io/?https://github.com/sqjin/CellChat/blob/master/tutorial/Comparison_analysis_of_multiple_datasets.html). The specific analysis steps were as we reported previously.^[^ ^7^ ^]^

### Code Availability

No custom code was employed for data analyses in this study. Any additional information needed to reproduce the analyses reported in this paper is available from the corresponding author upon request.

### Mice

H11‐Dmp1‐iCre mice (strain No. T004830), Sema5a flox mice (strain No. T026002) and Plxna1 flox mice (strain No. T019235) were purchased from GemPharmatech (Nanjing, China). Lyz2‐Cre mice were kindly provided by Prof. Chang Jiang (ShanghaiTech University). The Rosa26‐floxed stop‐tdTomato reporter mice were kindly provided by Prof. Weiguo Zou (Shanghai Sixth People's Hospital). Sema5a^fl/fl^ mice were crossed with Dmp1‐Cre+ mice to generate Sema5a‐CKO (Dmp1‐Cre^+^; Sema5a^fl/fl^) and control (Dmp1‐Cre^+^; Sema5a^+/+^) mice. Plxna1^fl/fl^ mice were crossed with Lyz2‐Cre+ mice to generate Plxna1‐CKO (Lyz2‐Cre^+^; Plxna1^fl/fl^) and control (Lyz2‐Cre^+^; Plxna1^+/+^) mice. The Rosa26‐floxed stop‐tdTomato reporter mice were crossed with Dmp1‐Cre+ mice to generate mice with Ocy‐specific tdTomato expression (Dmp1‐Cre^+^;tdTomato^+/+^). Littermate controls were used for all studies. Animal experiments were approved by the Animal Research Committee of the Shanghai Sixth People's Hospital (No. 2023‐0228). All animals were housed in Shanghai Sixth People's Hospital. The construction strategy for H11‐Dmp1‐iCre mice and the promoter sequence of mouse Dmp1 were referenced from the previous study.^[^ ^49^, ^50^ ^]^ The details were shown in supplementary file 1. Potential off‐target sites were predicted using *CRISPOR*, and three potential off‐target sites with higher scores were amplified by PCR followed by sequencing. Primers’ sequences used in the PCR for off‐target detection were shown in Supplementary Table S1 (Supporting Information). Sequencing primer sequences were shown in Supplementary Table S2 (Supporting Information).

### Flow cytometry and Cell Sorting

Primary osteocytes were extracted from Dmp1‐Cre^+^; tdTomato^+/+^ mice, following the specific extraction protocols previously reported.^[^ ^51^ ^]^ In brief, after euthanizing the mice, femurs were harvested, and the cells in the bone marrow cavity were removed. The bone tissue was subjected to repeated digestion with type I collagenase, type IV collagenase and EDTA solution. Following the collection of cells, a blocking solution was applied, and the cells were incubated with the primary antibody for 30 min at 4 degrees Celsius. Subsequently, the cells were washed with phosphate‐buffered saline (PBS) and underwent cell sorting.

### Western Blotting

For the preparation of total protein extracts, cells were washed and lysed according to standard procedures as previously described16. The protein concentration is determined by BCA assay (Thermo Fisher Scientific). Equal amounts of protein were loaded onto an SDS‐PAGE gel for separation based on molecular weight. After electrophoresis, the proteins are transferred from the gel to polyvinylidene difluoride (PVDF) membrane. The membrane was blocked with 5% nonfat dried milk and incubated with primary antibody overnight at 4 °C. After primary antibody incubation, a secondary antibody was applied. Bands were visualized using enhanced chemiluminescence on an imaging system (Tanon, Shanghai, China). Relative protein levels were quantified using ImageJ software (version 1.8.0; National Institutes of Health, Bethesda, MA, USA). Information on the antibodies and reagents used in this study is listed in Supplementary Table S3 (Supporting Information).

### Cell Culture Studies

Primary bone marrow‐derived macrophages (BMDMs) were generated in vitro by flushing bone marrow from mouse tibias and femurs. Osteocyte–osteoclast coculture were performed as previously described.^[^ ^52^ ^]^ Briefly, for establishment of osteocyte–osteoclast direct coculture system, osteocytes were seeded at 1 × 10^4^ cells per well on 6‐well plates for 24 h. BMDMs were subsequently seeded at 1 × 10^5^ cells per well on 6‐well plates in α‐MEM containing 10% FBS and 30 ng/ml M‐CSF for 7 days. MC3T3‐E1 cells were provided by The Cell Bank of Type Culture Collection of the Chinese Academy of Sciences. MC3T3‐E1 cells were differentiated in osteogenic induction medium.

### Scanning Electron Microscopy

The cells were fixed for 1 h in a freshly prepared standard FAA fixative solution containing 5 mL of 37% formaldehyde, 5 mL of glacial acetic acid, 63 mL of anhydrous ethanol, and 27 mL of double‐distilled water, followed by sequential dehydration in a graded ethanol series of 70%, 80%, 90%, 95%, and 100% anhydrous ethanol, with each concentration step lasting 5 min. The cells were then dried via critical point drying before visualizing by scanning electron microscopy.

### TRAP Staining

Osteoclasts were washed twice with PBS, fixed with 4% paraformaldehyde, and stained using a TRAP assay kit (TaKaRa Bio, Inc., Shiga, Japan) according to the manufacturer's instructions. Osteoclasts were then observed using a microscope. TRAP‐positive multinucleated cells with nuclei more than 2 were considered osteoclasts.

### Immunofluorescence Assay

Cells were washed twice with PBS and fixed with 4% paraformaldehyde. Following fixation, the cells were permeabilized with Triton X‐100 and then incubated with primary antibody overnight at 4 °C. After washing away unbound antibodies, a secondary antibody conjugated to a fluorescent dye is applied. The cells were observed under a confocal microscope.

### Bone resorption Pit Assessment

Osteocytes and BMDMs were cocultured on bovine bone slides for 7 days. Osteocytes and BMDMs were removed from bone slides using sodium hypochlorite solution. The bone slides were dried, sprayed with gold film, and then observed using scanning electron microscopy (SEM). Bone resorption areas were quantified using ImageJ software (version 1.8.0; National Institutes of Health, Bethesda, MA, USA).

### Dual‐Energy X‐Ray Absorptiometry (DXA)

The femur Bone mineral density (BMD) and total BMD (skull excluded) were measured with dual X‐ray absorptiometry (DXA, iNSiGHT VET DXA; OsteoSys, Seoul, Korea) after 8 weeks of treatment. The data obtained from the DXA machine underwent analysis using specific software to calculate BMD values, which were reported as grams of mineral per square centimeter (g cm^−^^2^).

### Enzyme‐Linked Immunosorbent Assay (ELISA)

The serum C‐terminal telopeptide of type I collagen (CTX‐1) levels were assessed using an ELISA kit (Kenuodi Biotechnology Co., Ltd., Quanzhou, China) according to the manufacturer's instructions. The optical density of each well is measured using a spectrophotometer. The results are compared against a standard curve generated from known concentrations of the analyte.

### Microcomputed Tomography (μ‐CT) Analysis

The mouse femurs were harvested and scanned using Bruker SkyScan Micro‐CT (SkyScan 1276, Allentown, PA, USA). Regions of interest were analyzed (1.5 mm above the growth plate of the distal femur). Bone volume per tissue volume (BV/TV), trabecular number (Tb.N), trabecular separation (Tb.Sp), trabecular thickness (Tb.Th) and cortical thickness (Ct.Th) were generated to evaluate trabecular morphometry using CTan software (Skyscan). Analysis Reconstructed scans were visualized via CTvox (v3.3.0),

### ALP and Alizarin red S (ARS) Staining

Osteoblasts were washed twice with PBS and fixed with 4% paraformaldehyde. ALP staining was performed using a BCIP/NBT alkaline phosphatase color kit (Beyotime) according to the manufacturer's instructions. ARS staining was performed to measure osteogenic differentiation. The fixed osteoblasts were stained with ARS staining solution (Beyotime) for 30 min. Osteoblasts were observed under a microscope. The positive area of ALP and ARS staining were quantified using ImageJ software.

### Reverse Transcription Quantitative Polymerase Chain Reaction (RT‐qPCR)

Total RNA from the cells were extracted using an RNA extraction kit (Qiagen, Germany). Following extraction, the RNA concentration and integrity are assessed using spectrophotometry. Once the RNA is verified, single‐stranded cDNA was synthesized from extracted RNA using a reverse transcription master mix (Takara, Shiga, Japan). Quantitative RT‐PCR was performed using the SYBR green qPCR master mix (EZBioscience, Roseville, USA). Relative gene expression was normalized to GAPDH, and primers for other genes were listed in Table S4 (Supporting Information).

### Co‐Immunoprecipitation (Co‐IP)

Co‐IP was performed following the protocol with the Protein A+G Agarose system (Beyotime). Cells were lysed with a diluted lysis buffer. Ten percent of the lysate was reserved as input and incubated overnight with either anti‐Plxna1 antibody or control anti‐mouse IgG. The resulting immunocomplexes were incubated with protein A/G agarose beads, washed three times with lysis buffer, and eluted using SDS‐PAGE loading buffer (1×) by boiling for 10 min at 100 °C. Following this, the samples were analyzed using Western blot.

### Transcriptome Sequencing

Transcriptome sequencing was performed by OEBiotech (Shanghai, China). Total RNA was extracted from cells and the transcriptome was sequenced using the Illumina sequencing platform. Differentially expressed genes (DEGs) were analyzed with the DESeq2 package (1.34.0). Gene expression profiles of GSE 230 665 were obtained from the Gene Expression Omnibus (GEO) database.

### Histology and Immunohistochemistry

Femurs were collected, fixed, decalcified, embedded and cut in 10 µm sections. The slices were stained with hematoxylin and eosin (H&E). For IHC analysis, slices were mounted on slides, baked, dewaxed with xylene and hydrated with a gradient ethanol series. Primary antibodies specific to the target antigen are then applied, followed by incubation with secondary antibodies. Finally, the samples were stained with 3,3′‐diaminobenzidine tetrahydrochloride at room temperature for 25 s and counterstained with hematoxylin at room temperature for 5 min. The slides were observed under a microscopy.

### Statistical Analysis

All experiments were conducted a minimum of three times. The data are presented as the means of triplicate biological replicates ± standard error. For statistical comparisons between two groups, an unpaired two‐tailed Student's *t*‐test was utilized. In cases involving more than two experimental groups, ANOVA was performed followed by the post hoc Tukey–Kramer test. A P value of less than 0.05 was regarded as statistically significant.

## Conflict of Interest

The authors declare no conflict of interest.

## Supporting information



Supporting Information

Supporting Information

Supporting Information

Supporting Information

Supporting Information

Supporting Information

## Data Availability

Research data are not shared.
