# Supplementary material for: Identification and Mechanisms of Osteocyte Subsets Involved in the Pathological Progression of Osteoporosis
Source: Adv Sci (Weinh). 2025 Nov 18;13(5):e13427. doi: 10.1002/advs.202513427 (PMC12850396; doi:10.1002/advs.202513427)
Supplement: Supplementary file 1 — Supporting Information [file ADVS-13-e13427-s001.docx]

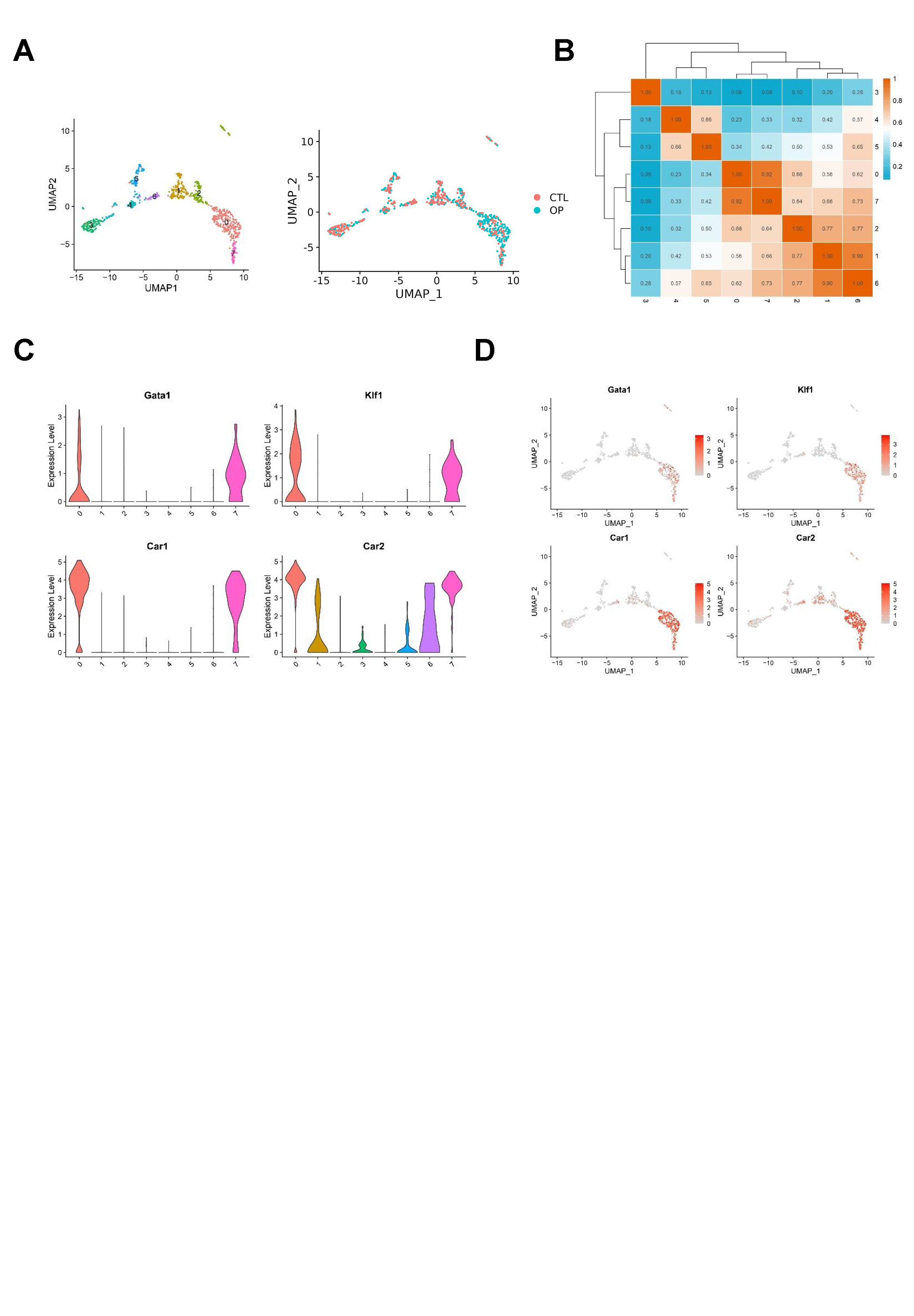


**Supplementary Fig. 1** The identification of osteocyte subsets. (A) UMAP analysis of 8 osteocyte group subsets. (B) Heat map represents Pearson correlations of 8 subsets with each other. (C) Violin plots of Gata1, Klf1, Car1, and Car2 in 8 subsets. (D) Feature plots of Gata1, Klf1, Car1, and Car2 in 8 subsets.


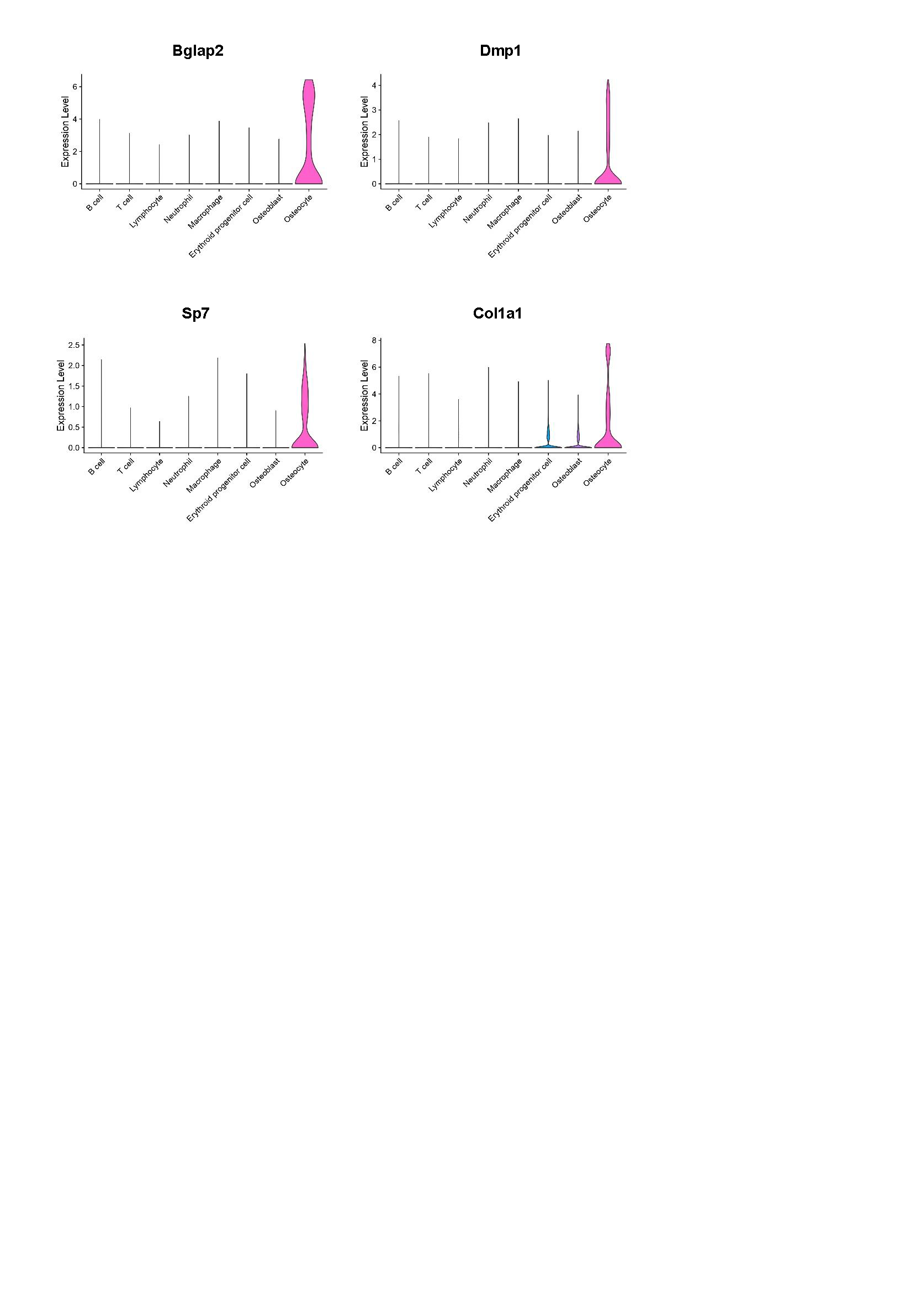


**Supplementary Fig. 2** Violin plot showing the expression of well-established osteocyte markers, including Sp7, Col1a1, Bglap2, and Dmp1 in identified cell types.


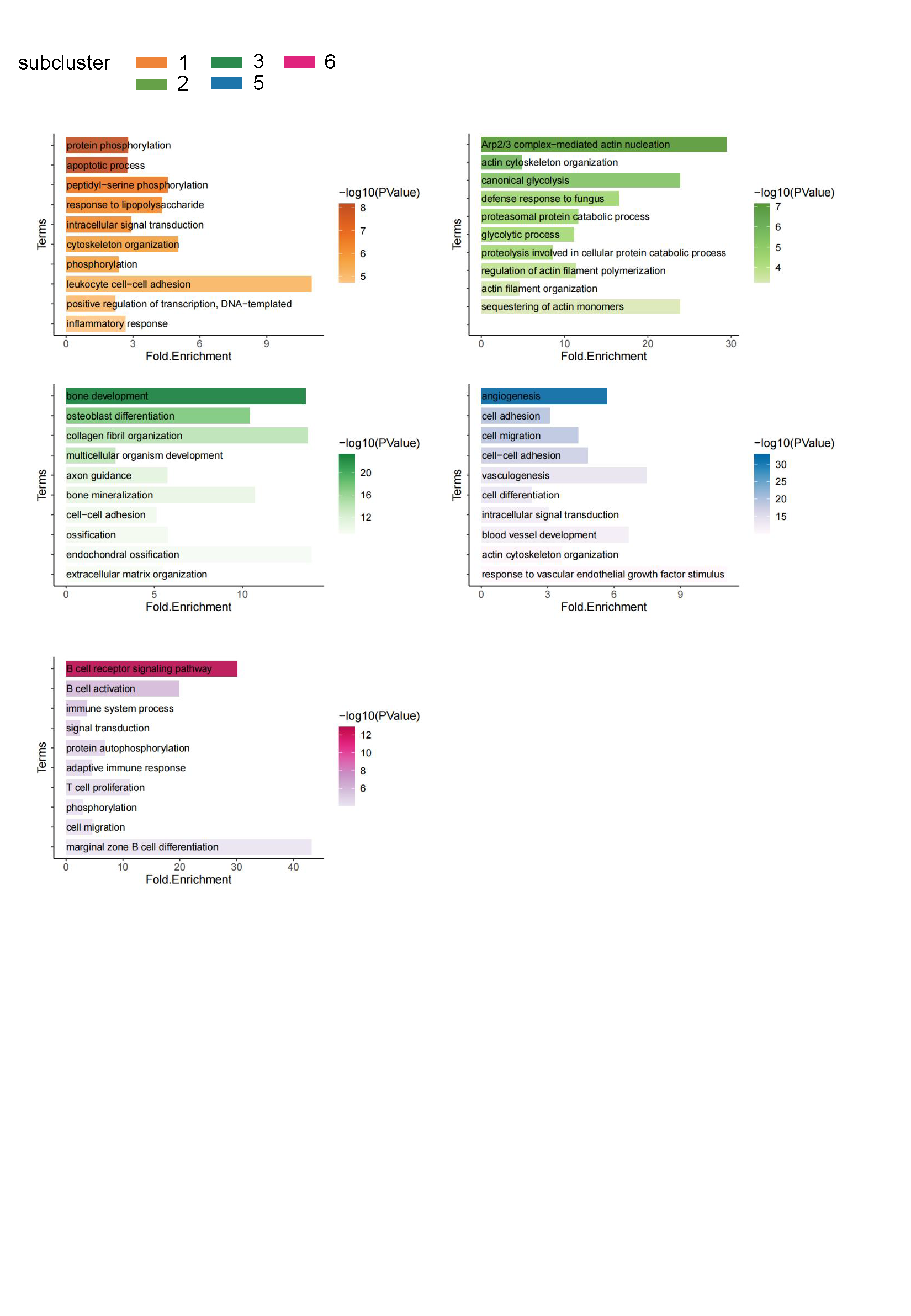


**Supplementary Fig. 3** The GO analysis results based on DEGs between the different osteocyte subsets (cluster 0, cluster 1, cluster 2, cluster 3, cluster 5, cluster 6, cluster 7)


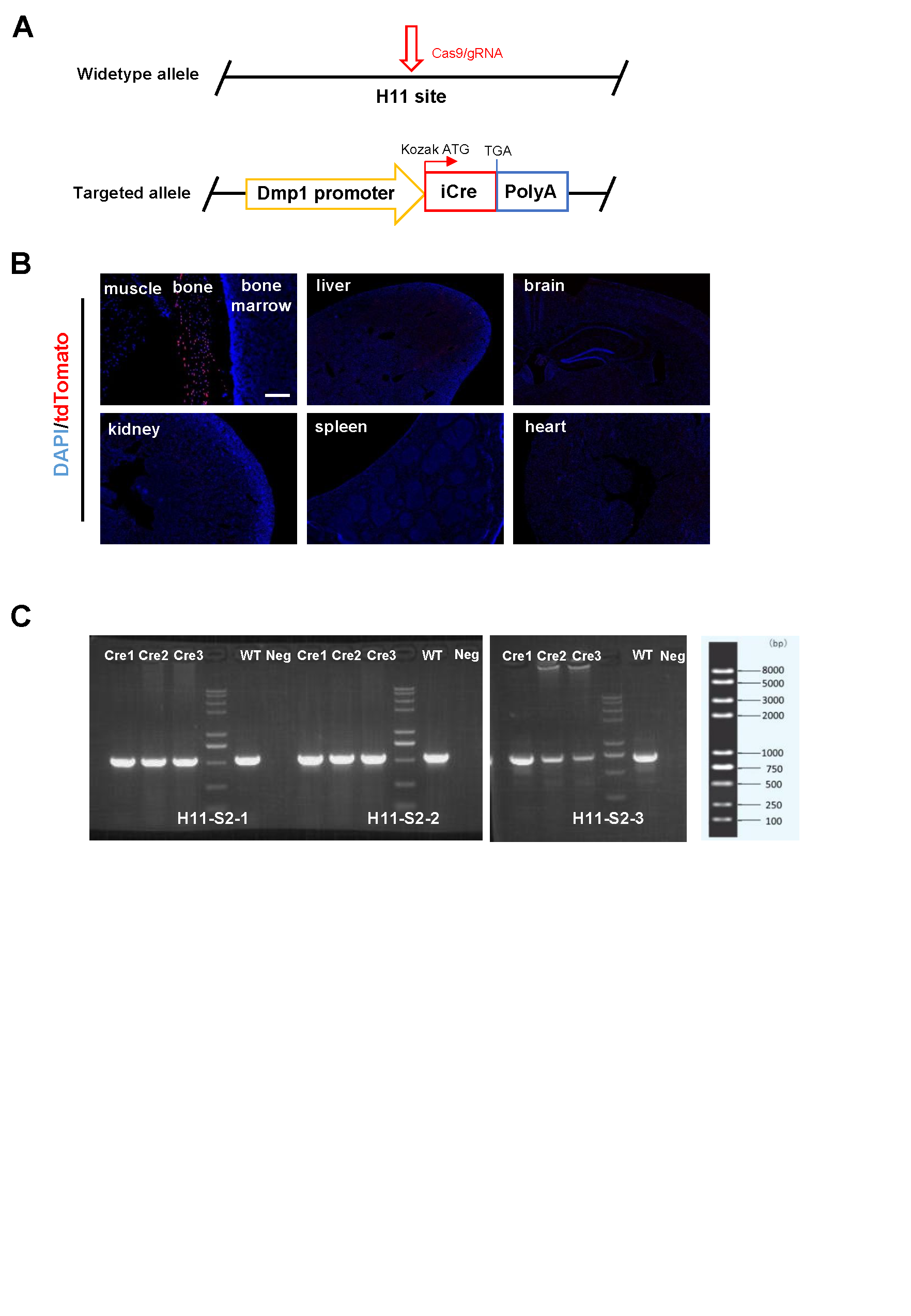


**Supplementary Fig. 4** Construction and verification of Dmp1-cre mice. (A) The schematic representation of *the* *strategy* for constructing Dmp1-Cre mice. (B) Red fluorescence (tdTomato) in different tissues was observed under a confocal microscope (scale bar, 500 μm). Red fluorescence was only observed in osteocytes embedded within the bone matrix. (C) The results of PCR revealed that no off-target effects were detected at any of the off-target sites. Cre: Dmp1-Cre^+^ mice.

**
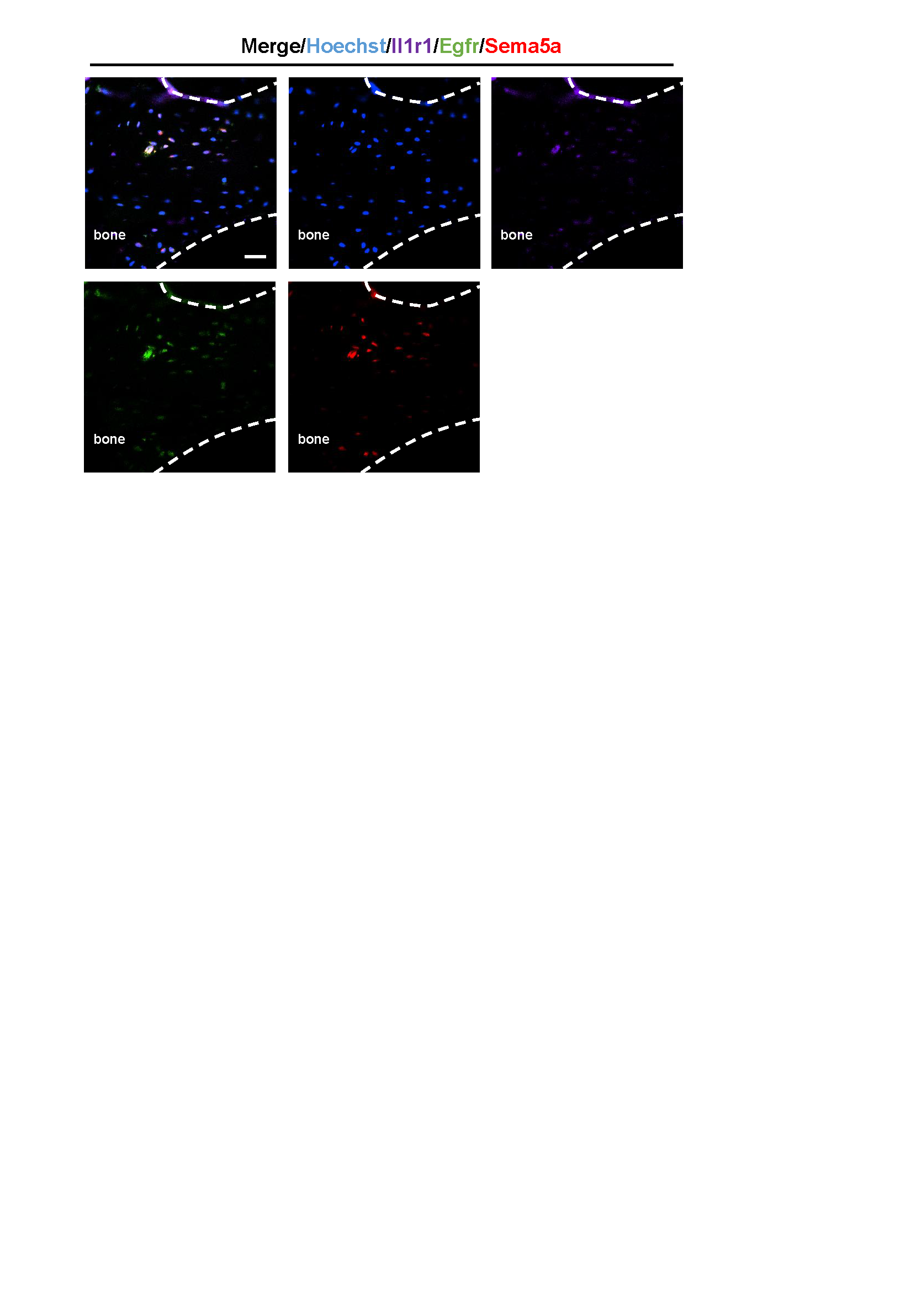
**

**Supplementary Fig. 5** Representative images of immunofluorescence co‐staining in mice (wt). The results of immunofluorescence co‐staining confirmed the existence of BHR-Ocys (Il1r1, purple; Egfr, green; Sema5a, red; nuclei, blue; scale bar, 20 μm).

**
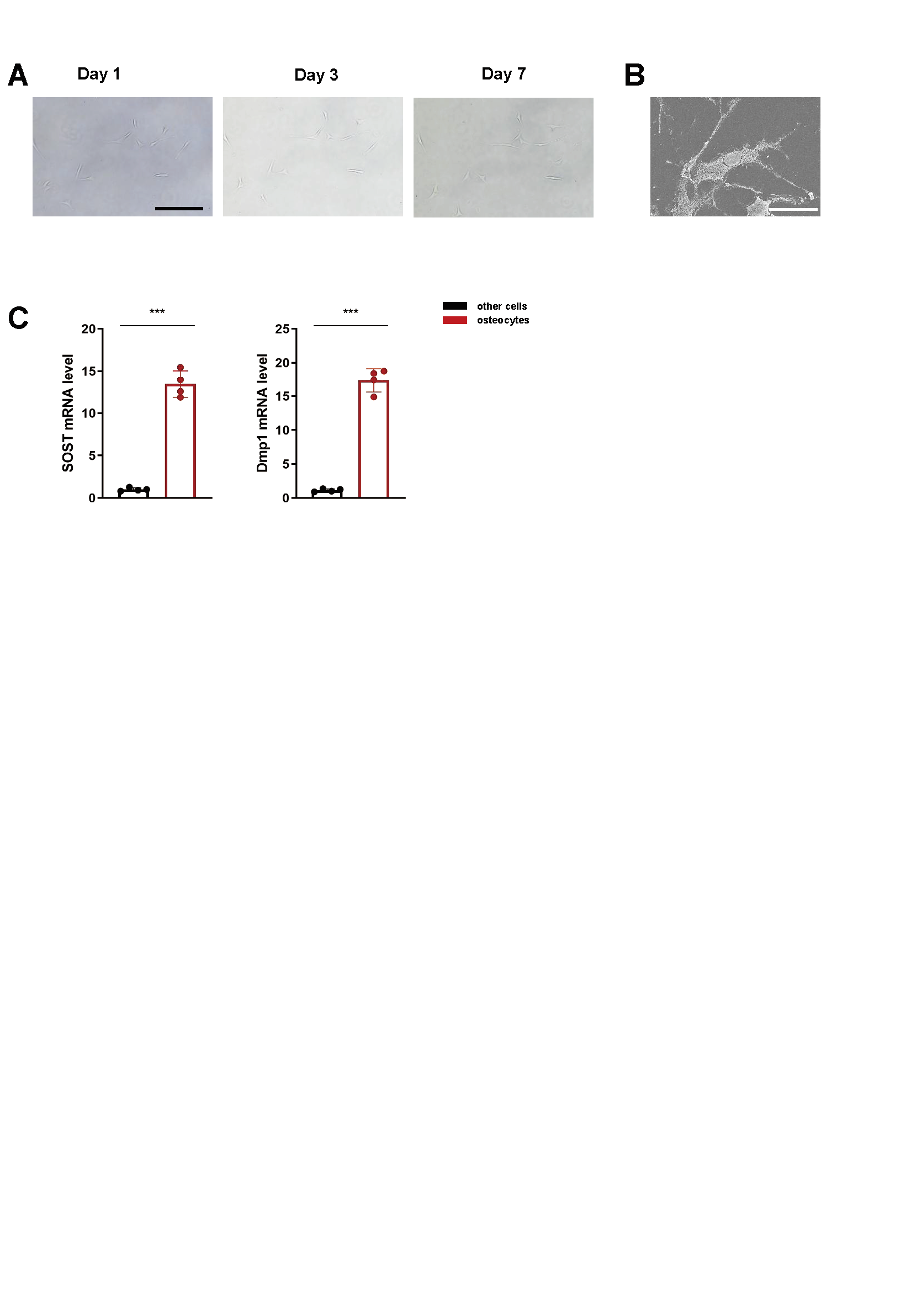
**

**Supplementary Fig. 6** Validation of isolated cell types. (A) Microscope images of cells on day 1, day 3, and day 7 after complete attachment (scale bar, 200 μm). Cells did not proliferate during cultivation. (B) Representative scanning electron microscopy pictures of showed that the isolated cells exhibited the characteristic dendritic morphology of osteocytes (scale bar, 50 μm). (C) The results of qPCR showed that the expression levels of SOST and Dmp1 in the target cells were significantly higher than in other cells. (n = 4/group). Data are represented as the mean ± SD. ***P < 0.001. Statistical analysis employed two-tailed unpaired Student’s t-test.


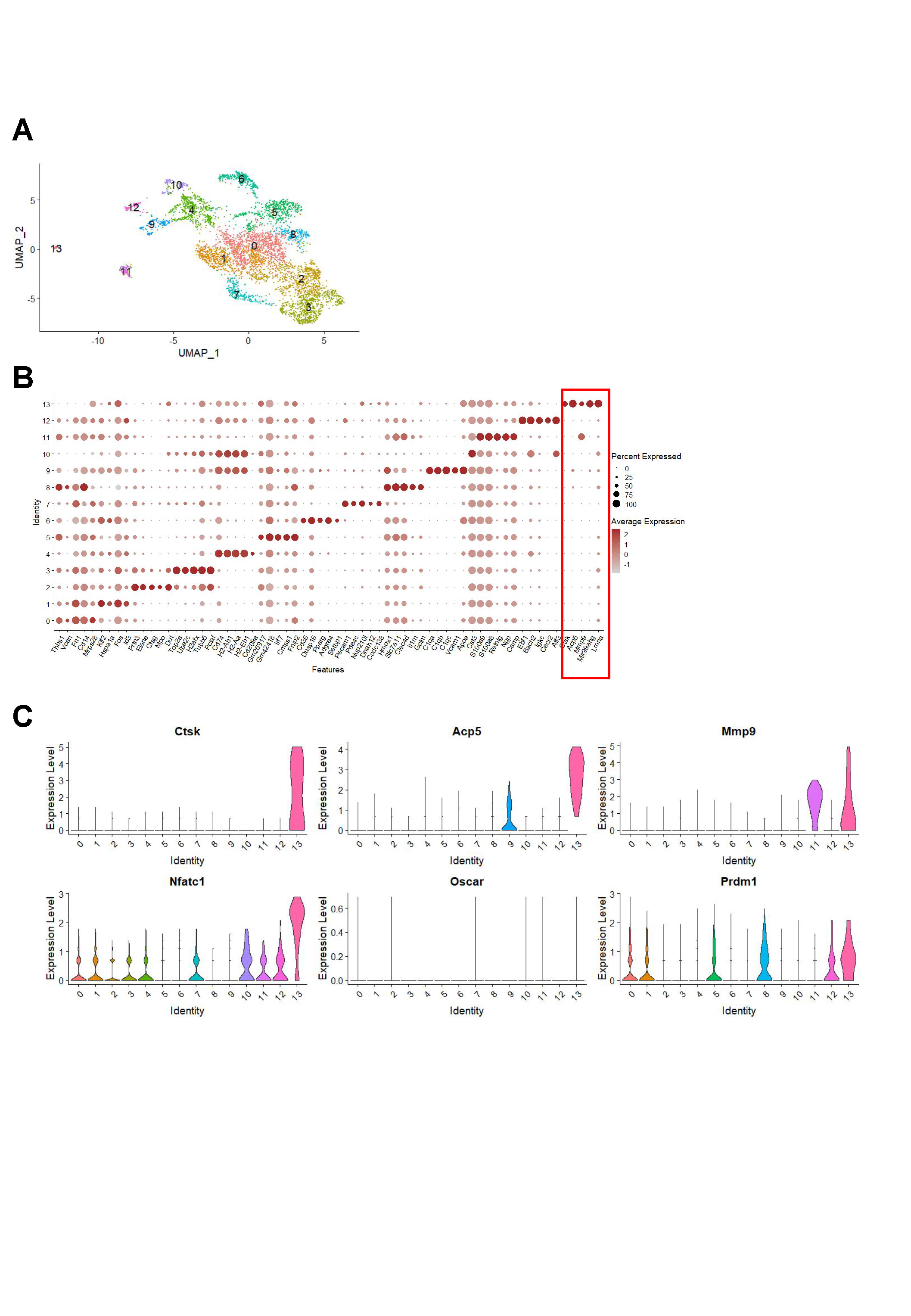


**Supplementary Fig. 7** Single-cell sequencing identified osteoclast cluster. (A) UMAP analysis of macrophage group subsets. (B) Dot plot showing the expression of specific signatures in macrophage group subsets. The red box indicated that osteoclast-related markers were significantly overexpressed in the cluster 12. (C) Feature plots of Ctsk, Acp, Mmp9, Nfatc1, Oscar, and Prdm1 in macrophage group subsets.


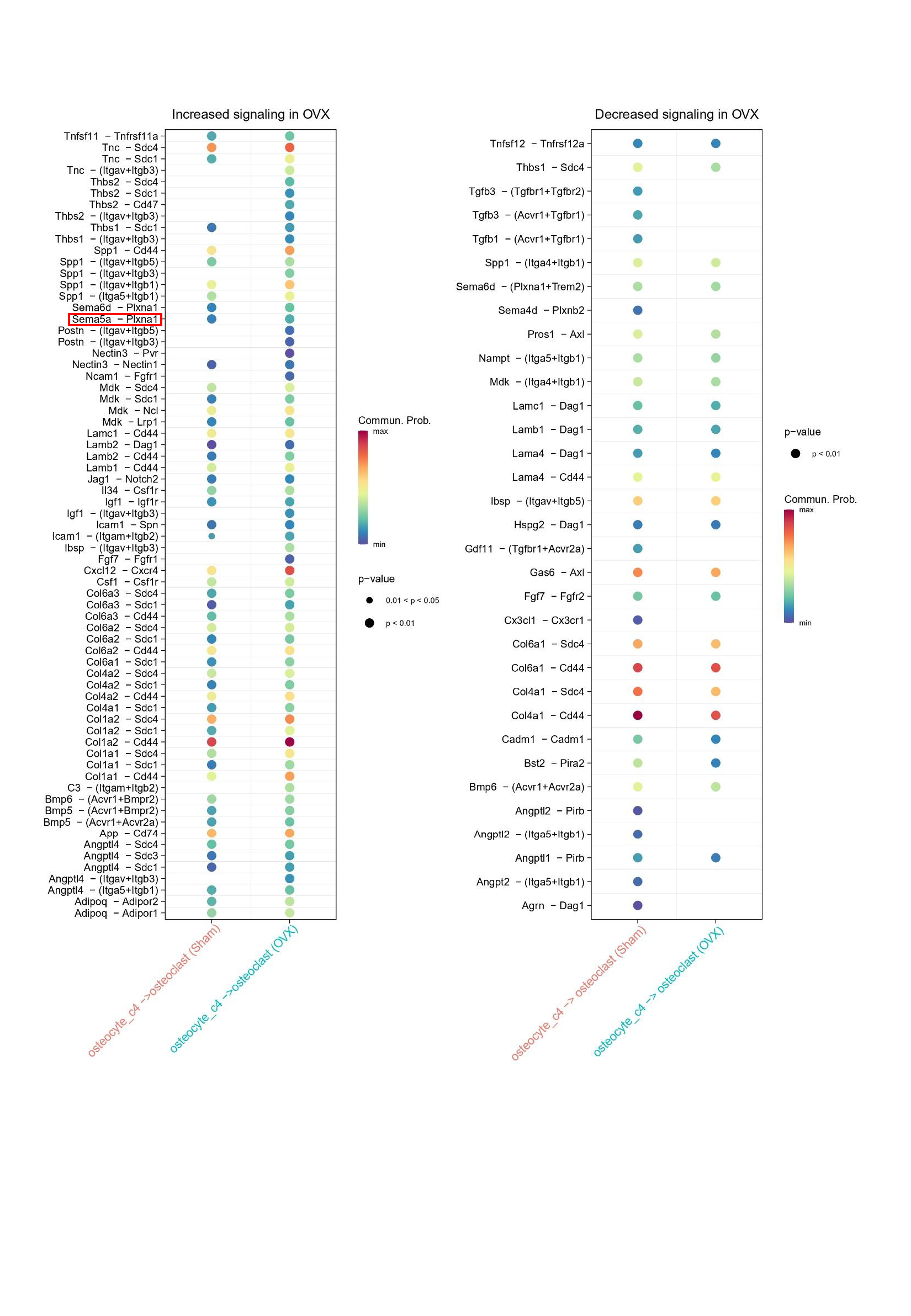


**Supplementary Fig. 8** The results of receptor-ligand analysis showed that the Sema5a-Plxna1 axis is an important receptor-ligand pair for communication between BHR-Ocys and osteoclasts.


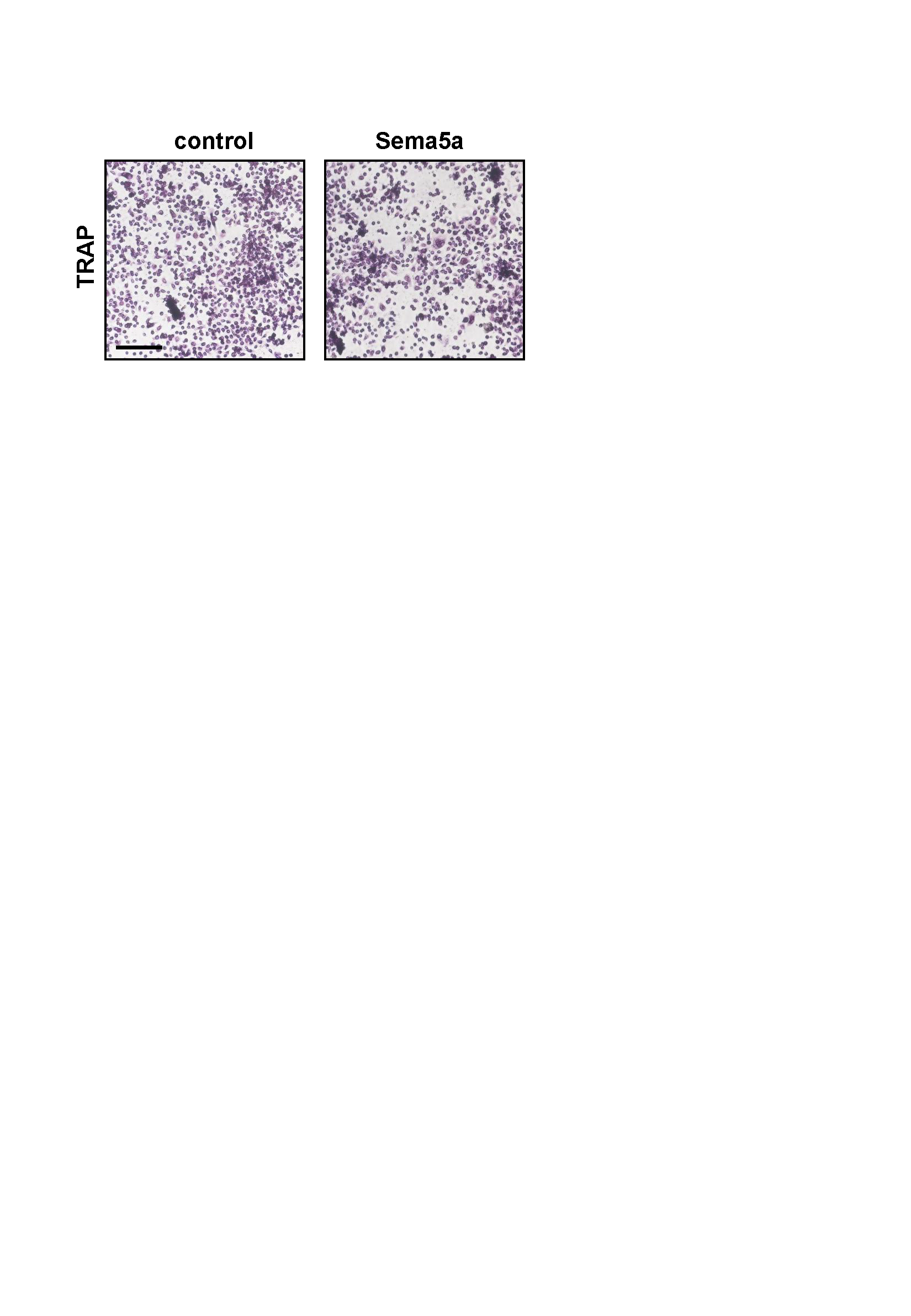


**Supplementary Fig. 9** Sema5a was unable to independently induce the differentiation of BMDMs into osteoclasts in the absence of RANKL. Representative images of TRAP staining of BMDMs. BMDMs were treated with 10 μg/mL Sema5a and 10 ng/mL M-CSF for 5 days.


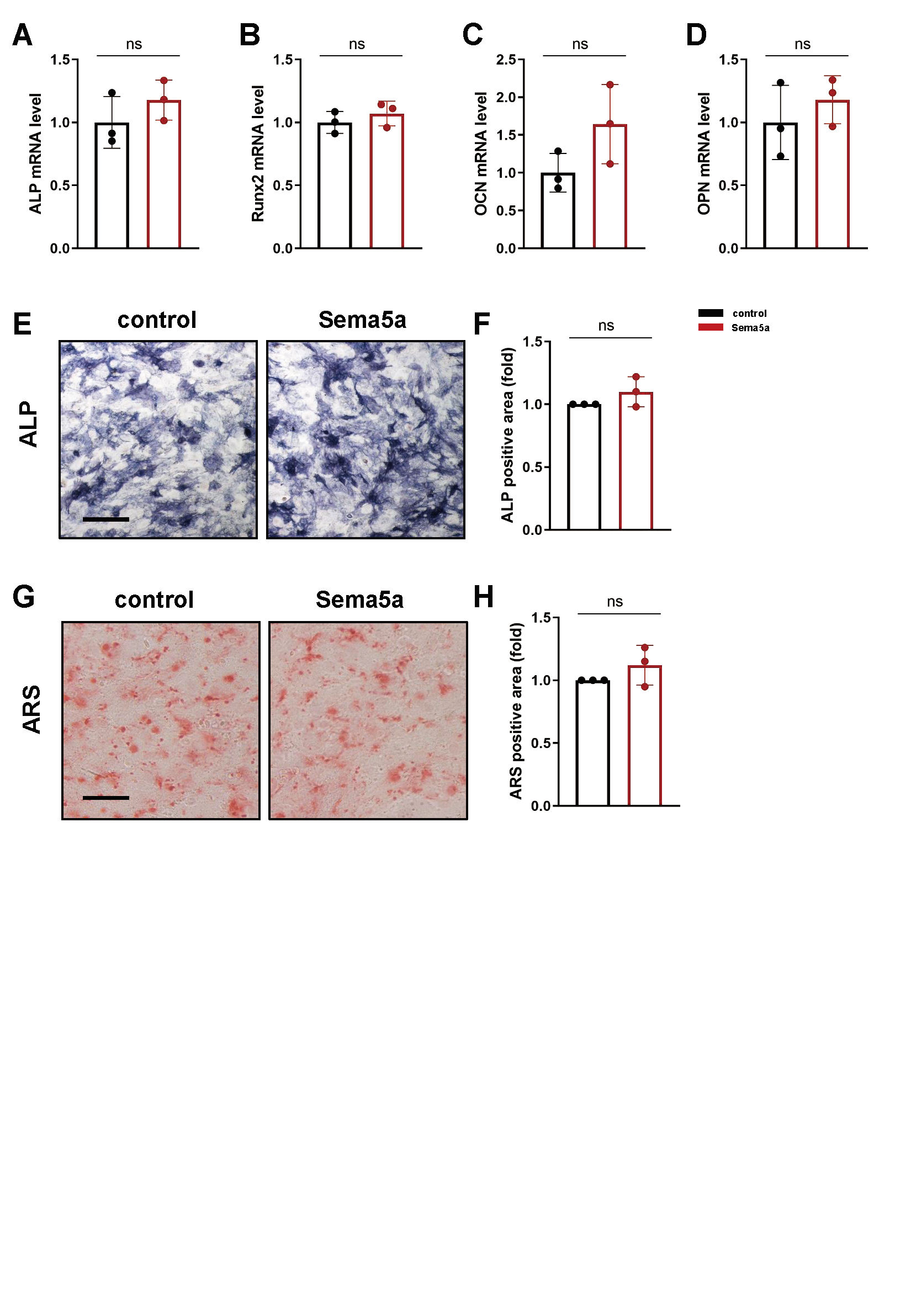


**Supplementary Fig. 10** Sema5a did not exert a significant effect on the differentiation and mineralization capacity of osteoblasts. (A) ALP, (B) Runx2, (C) OCN, and (D) OPN mRNA levels in MC3T3-E1 osteoblasts were assessed by RT-qPCR. MC3T3-E1 osteoblasts were treated with or without 10 μg/mL Sema5a and cultured with osteogenic-inducing media for 7 days (n = 3/group). (E) Representative images of ALP staining in osteoblasts (scale bar, 200 μm). (F) Quantitative analysis of the ALP-positive area normalized to the control group (n = 3/group). (G) Representative images of ARS staining in osteoblasts (scale bar, 200 μm). (H) Quantitative analysis of the calcium deposition normalized to the control group (n = 3/group). Data are represented as the mean ± SD. ns = not significant. Statistical analysis employed two-tailed unpaired Student’s t-test


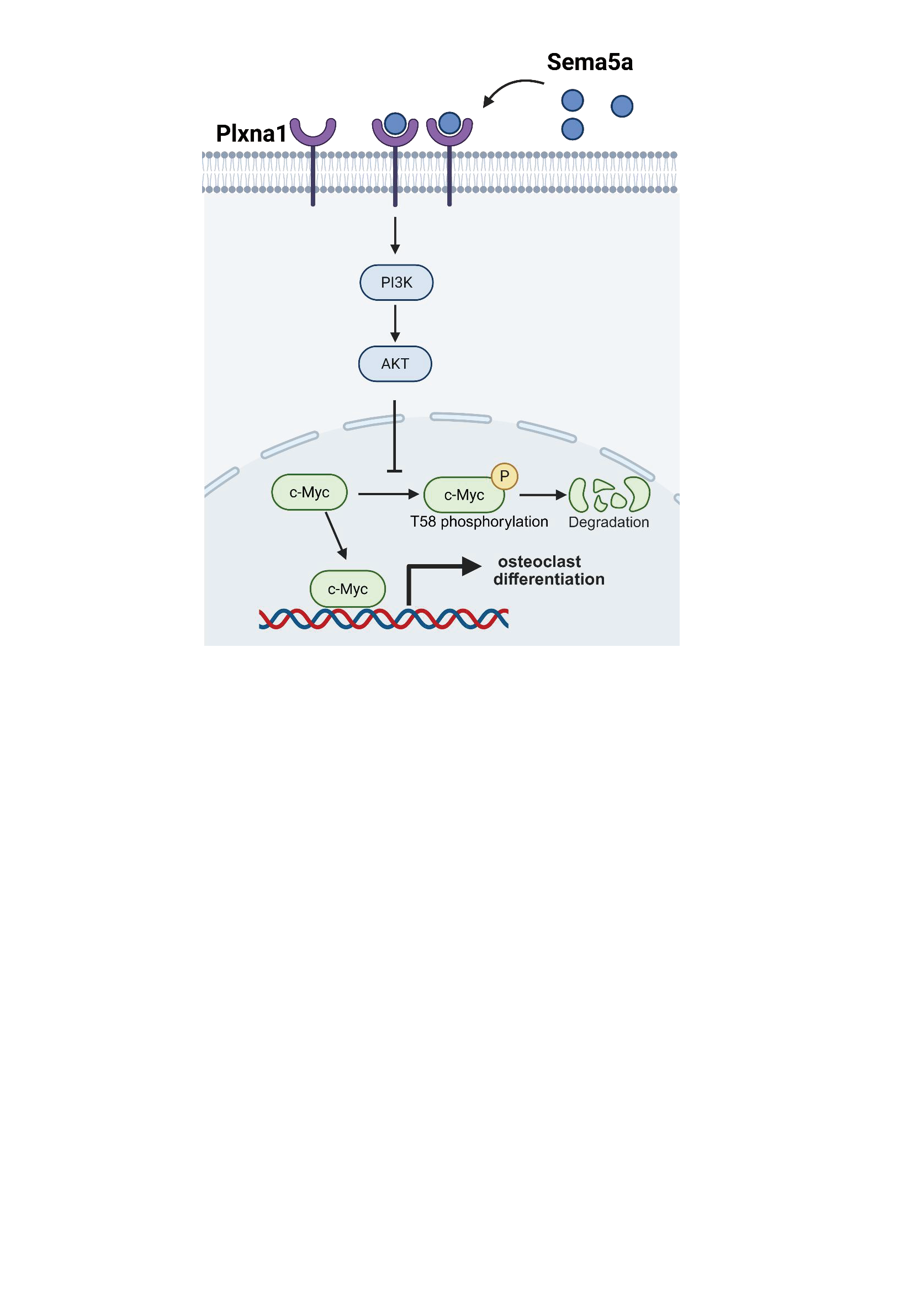


**Supplementary Fig. 11** Schematic illustration of the PI3K/AKT/c-Myc signaling pathway activated by Sema5a.


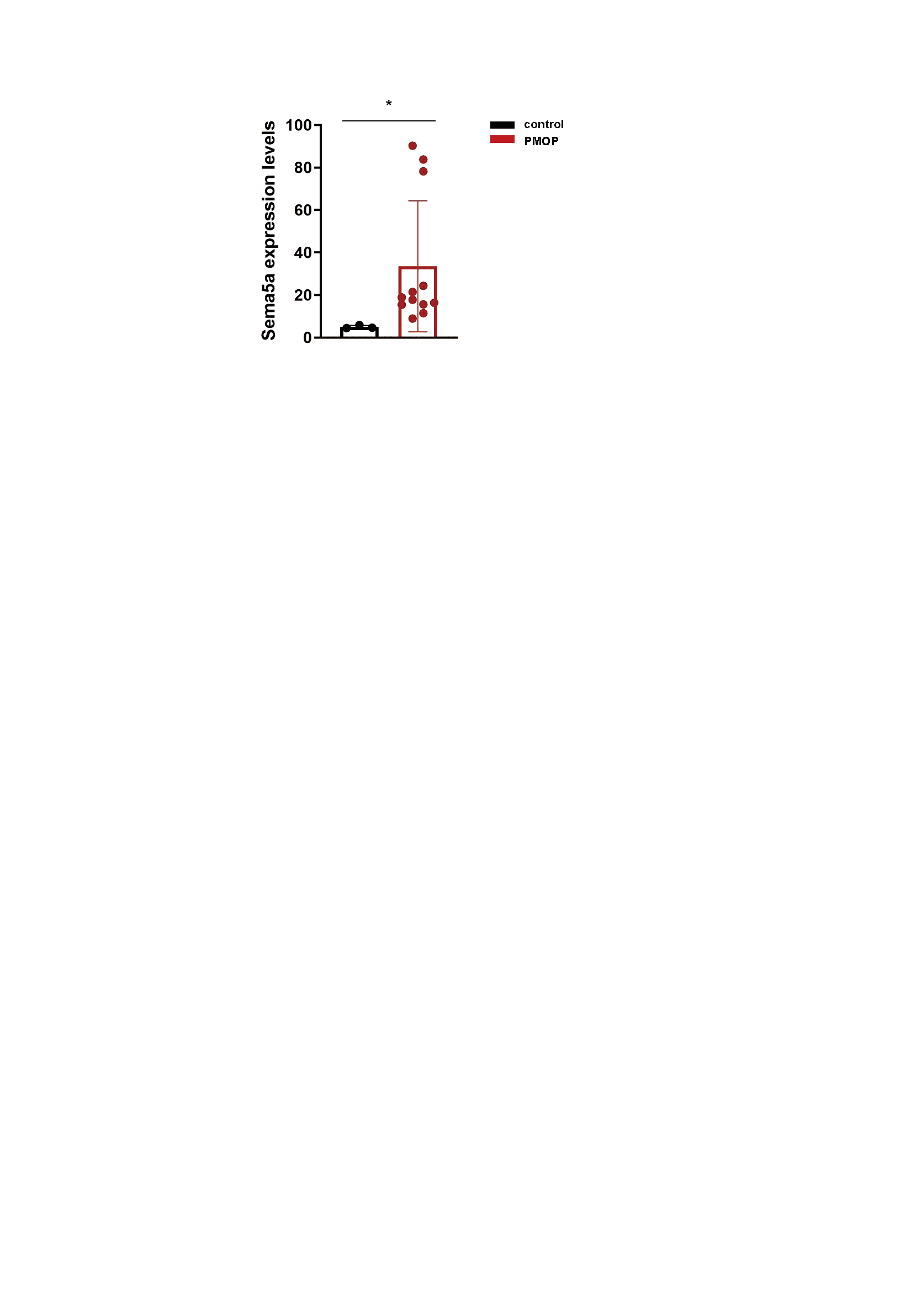


**Supplementary Fig. 12** Sema5a expression in bone tissues (femurs) of patients in different groups. The levels of Sema5a in bone tissues of PMOP patients were significantly higher than those in the control group.
