# Supplementary material for: Identification and Mechanisms of Osteocyte Subsets Involved in the Pathological Progression of Osteoporosis
Source: Adv Sci (Weinh). 2025 Nov 18;13(5):e13427. doi: 10.1002/advs.202513427 (PMC12850396; doi:10.1002/advs.202513427)
Supplement: Supplementary file 2 — Supporting Information [file ADVS-13-e13427-s005.pdf]

Supplementary Table 1. Primers' sequences used in the PCR for off-target detection

| No. | Forward (5'-3') | Reverse (5'-3') |
|---|---|---|
| H11-S2-1 | CCAATCTGTCTAGTCCAGGTTTCCA | TGGCTTTGTAAAGTTGTCTGGGC |
| H11-S2-2 | GAGGGCAGTAAGGTGGTGAA | CCATAGATGCGCTTGTAGGC |
| H11-S2-3 | ACCCAGAAGACTGTGGATGG | TTCAGCTCAGGGATGACCTT |
