# Supplementary material for: Identification and Mechanisms of Osteocyte Subsets Involved in the Pathological Progression of Osteoporosis
Source: Adv Sci (Weinh). 2025 Nov 18;13(5):e13427. doi: 10.1002/advs.202513427 (PMC12850396; doi:10.1002/advs.202513427)
Supplement: Supplementary file 3 — Supporting Information [file ADVS-13-e13427-s002.pdf]

Supplementary Table 2. Sequencing primer sequences:

Primer Name	Forward (5'-3')	Decription
H11-S2-tF1	CCAATCTGTCTAGTCCAGGTTTCCA	H11-S2-1
H11-S2-tR2	AAAGGTCCCGTCCAACACTGTGT	H11-S2-2
H11-S2-tR3	GCTTTGCCTGCACTTAACCACTTA	H11-S2-3
