# Supplementary material for: Identification and Mechanisms of Osteocyte Subsets Involved in the Pathological Progression of Osteoporosis
Source: Adv Sci (Weinh). 2025 Nov 18;13(5):e13427. doi: 10.1002/advs.202513427 (PMC12850396; doi:10.1002/advs.202513427)
Supplement: Supplementary file 4 — Supporting Information [file ADVS-13-e13427-s006.pdf]

Supplementary Table 3. The details of antibody information.

Antibody	Company	Catalog Number
RANKL	Proteintech	23408-1-AP
Spp1	Proteintech	30200-1-AP
Csf-1	Abcam	ab233387
β-tubulin	Proteintech	10094-1-AP
Il1r1	ABclonal	A25859
Egfr	Thermofisher	MA5-13070
Sema5a	R&D Systems	AF5896
Plxna1	R&D Systems	AF4309
c-Myc	Abcam	ab32072
p-c-Myc (T58)	Abcam	ab185655
PI3K	Cell Signaling Technology.	4249T
p-PI3K	Cell Signaling Technology.	17366T
AKT	Cell Signaling Technology.	9272S
p-AKT	Cell Signaling Technology.	4060T
