# Supplementary material for: Identification and Mechanisms of Osteocyte Subsets Involved in the Pathological Progression of Osteoporosis
Source: Adv Sci (Weinh). 2025 Nov 18;13(5):e13427. doi: 10.1002/advs.202513427 (PMC12850396; doi:10.1002/advs.202513427)
Supplement: Supplementary file 5 — Supporting Information [file ADVS-13-e13427-s003.pdf]

Supplementary Table 4. Primers’ Sequences Used in the Real-Time PCR (5' - 3')

Gene	Forward (5’-3’)	Reverse (5’-3’)
ALP	CCAACTCTTTTGTGCCAGAGA	GGCTACATTGGTGTTGAGCTTTT
OCN	GAGGGCAGTAAGGTGGTGAA	CCATAGATGCGCTTGTAGGC
Runx2	TGTCCGCCACCACTCACTACC	GGGAACTGATAGGATGCTGACGAAG
Dmp1	ATCCCAGGGCTTGGAGAGTA	ACATCTTTGGCGTCATAGGG
SOST	GGTGATTTGGCTGGGTC	TGTGGTCACTATTTGCCTGT
GAPDH	ACCCAGAAGACTGTGGATGG	TTCAGCTCAGGGATGACCTT
