# Supplementary material for: Identification and Mechanisms of Osteocyte Subsets Involved in the Pathological Progression of Osteoporosis
Source: Adv Sci (Weinh). 2025 Nov 18;13(5):e13427. doi: 10.1002/advs.202513427 (PMC12850396; doi:10.1002/advs.202513427)
Supplement: Supplementary file 6 — Supporting Information [file ADVS-13-e13427-s004.zip › Supplementary File 1.pdf]

Promoter Sequence of Mouse Dmp1 (14108bp):

GTGGGCACTGTCTGTGTAATAATTACTGTTGTCAGAGAAGCCCTTGATGGCT
ATGCTTCTCCTCATCGAGTTCAGCTACCTCAAAGTGGCAAGCTACTCACCT
GACGTAACAGCCAGGTGAACTAGAAACCCATTTATCTTCCAGGTTCTTATAT
TCCTCTGGCATGTTGATGGTGCAGCTGATGAATATTTTTTCCATCAACATAA
ACTAGGCTTATGGGAATATAAATATGTGCCTAATCTGTGTTTTTTCAAATAAA
TATAATTTGTTCTTTTCACACGCTACAAGGAAAAGTATGAATAATTGAGAAA
ACACACTCCACCAGATTTGTGTGCAGACAAGCACCTGAGATCCAGTGGGC
TCCGAGGTGCTGCGATCAGCTTGTCCCTCTGTCTGTCTGCACTTCTCTGTTA
TTGCTAGCATACACATTCAAGCTTAGACAGACCTCCACGTGGTTGATCTCAT
TCTTAAAACCCACTTCTCAGCTCAGGGAAGGTCAGGAGCCTAAAGCAGGA
ACGTGAACAGCTCTGTCGTAATTTGGTCTAAGCATCATTTAAGGTTGGCAA
AGGAAACACATGGCTGGAATGGAAGCAACATCATTTATTTTTATTCTAGTAG
CAATTTTCTCGAGATCAGAACATTCTGAGGTCTCCATATTGGAAAAGAATCA
ATTTTTTTTTCTGCTAGATTTAAGCTCAACTTTTTCAAATAACCAATTTACGG
CATTGTAAATTCGAGAATAGACTCTAGGTAGCTGTAATTTTCAGATGTTTAA
AAAGAAAATTGGAAAAGTTCTTATATAGCTCCCAAGGGTCAAAGCCACCAC
CACTGCAACAGCAAAGATCAATTTAAGTGCAGGATGAGAAAAGGAAGGGT
TTCAAACTCTGAGATGTATGCTGCTCCCAAACTACTGTGGGCATGCGTTATG
GGGCAGCTCAGCAGGCAGGGTCTACAGTTGGGAATGGTTGTTGGTGGCTT
TCTCCTGAACAGCCTACTTCCCACCTCCTGACTGAGGACACCAGCCAGCCA
GTGCAGAGGACCTTTCCAATCTGATTTCTCAGCGACATAGTACTTTCATAAA
GACAGAAGACCCCTTACCTCTAATTTGGTTCAGTTTATACAGGAATGGCAG
GTGCCTCAGCTGTCTATGTATCTGAAAACCTTTGAGCGAATTAGCTGGTTAC
ACATCTTCACGCTAAGGTGACCAATAGCTCTCTGTTTTATTTTTCTAAGGGC
ACTGCAGATTCAAAGGTTCGGTTACGTGCTAGCCTTCAGTGTGTTGTAGTTT
TATTGTTTCCTTTGTCGCTGGTGAAGGCCATTGAAGCTGCCTCCCGCAACAA
TCGCCAGATTATGAACTTTCTTCTTCATCAAATCTGAATTCCCCTGTTCTTCA
ATCTTAAGATCACGAGTCTCAAAAAACACATTGGTCAAAAGAAGAGGAGC
AACTCAAAATAACGAAATCTCTTTATCCCACACTATATACACAACAGTTTTG
AGGTAACAATAATATCATGATCACTGGTATAATTAAGAACAGTCTAAATTACT
TTTGTGTAAATATTCTTCATTTCCACTTTCTATGCTGTAAAGCAGGAAAAGT
TTACATTAGTCTAGTCATTTTACTTACTGAATTTTATTCCTCTAGGGGTGCAA
CCCCTGAACAACCCCCTGTCTTTACATTTTCAGAGAGCTCTAAGCTGCTGC
TGCTGCTGCTGCTAGGCCTTGAATCTGTAACTGATTCTTTTGGCCAGTCAGA
GTTTGTCAGTTATCTGTTTGCCCTGCATCAATTACACTGCTTGAACTTGCTC
AGGGAGAGATATTCCAAGAAGATTCCTGGAGTAATGGCCTCCTCTAGCTCT
GTGCTTATCTGTGCTTAATGTTCTCCAGGGCATAAGGAAGACTTCCAAATAG

TGGCCAGATAAAGTTAAATTTCAGATTTCCTAAGAAGACTCAGACTTAATTT
TCTCAGGAAAAAAACATAAAATGAATCATAATATAGGAAGTCCCCAAGACT
GCAACACACAGAGACCAAAACCCAAAAGTCAGAGAGAGAAAGAGTGACT
ATAGCCATCGCCAGCTTTGCTGGAACTGTGTCCGGCAGCTGTGCTGGCCTT
GTGCCTCTCACTGTTTCCAGTGGGTATGGCTGTGCCAGAGTAAAAGCCAAA
TTATACTGGTCTATGAAAAGCACATACTAGTCAAATCATCACTAATGACATT
CTTTTCCATTTGTGCATCAAGGCCTTGCTTGGCTGTCACTAGAGAAGCTTCC
TCCGGCAGCAGATGGGAACCAATACAGAGACTCACAGCCAGACAATGTGC
AGACTGACAGACTTGTAACACCCACTCTTACATGGGATGTCTCCATCAAAT
CTCCACCCTCAGAGCTCAGGAAACCCAGAGAAGGAGACAGAAAGAGTGT
GAGAGCCACAGGAGATAAGAACACCAGGTAAGCAGGGCCCTCTACTTCAA
CAGGATCAAAGCTCATATGAAATTACAGAGGCCAAAGCAGTATGAACAGA
GTCTGCACCAGATGGGGCCTTAAAGATCAAAGAAGTGGACACAAGTCCCA
TTCCTAACCCAGAGGCATCTACAATTGATAACCTCTGCCAATGAAAGATTAA
TTTTTTCCCAAGGGAGTCTCACCTTAAGTCCACCCTTAAGGGTAGGATATAT
GATCAGCAGCAGATAGCCAACAGAAAATAAACTCGAGAGAATCTTTGGAG
GTTCCTTGTCTATTCATGTCATGTTTGTTTGTTTTATTGCATATATATAGATATA
GATAGATATAAATATAGATATACACACATATATATACATATATACATATATAATG
TTATATGTATTATATACAATATAAACACAATATATAACATGCAATATCTAACAT
GTGGTATGTAATATGTAAAACATACATATGTATATATAATATACATACATTTTAC
ACATATAAAATATATAAATATATATGTATTACATATATATATATGTATATGTGTGT
GTTTTCTTACCCTACCAGTTTTTTGGGTATGTATATATCATGACTTCCAGTTTA
GTGTTTTTATGGCATTCTTGAGTGTGCAAATGAGTGGGTCTTGCATCTATATC
CGTTTCTTGTGTTTTTCCCCTGGGCTTTTCTTCTCCTATTTGCTTTTTTTGTCC
TATTCCAATGTGTTACTTTTAGCTTAATTTAATTTTCTTATTATGCACTAGAAG
CATGTTTGATTTCTAATGAGAGACAGAAAGGGGGTGGATCTGGATGGGAGA
GGAGGTGTGGAGTAACTTGGGGTAGTAGAGGGAGGGAAATGGTAACAGAG
TACAGTACATAGGGAAAAATCTATTTTCAATAAAAGGAAAAAAAAAGAAG
AAGAAAGAAAAAAAATCTCACAAAGCTTGGGATGCAAGCCCAGCCATCTT
AGTATCAGGGATGCCGAGGCAGGATACATTTTCCAAGTTCAAGCGAGCCTG
GGCTACAGAGTGAGACTATGTATCAGAAAACAAAAGTAACAAGGGGGAAA
AAAAAGAGTGTTGTTGTACAGCCCTATGGGGAAGTAAAGGCAGGGGGGCC
AGGAAGAGTTATCTCAAGGTAGCTCTGTAAGTATTCAGTGAAGTCTAGCCT
CGGGTGGGTACAGGTGACCTCATTGGGAAATAAAAGAACCTGGTCAGTAA
GCTGGGAAGGCAATGGGCAGAAGTGGTATAGCAGACAACAATAGGCTCTG
GGTGTTTCTGAATCCATAGCCAGGGCCTCCTGTAGAGCCAGCAAGGCCACA
CCGCACAGGCCCTCTGTCTGCCCTGATCCCTCAGTCAGCTTGAGGGAGTGG
TTCCAGAGTTTTCCTTTCCTATCAGAATCCAGTGACTTTATTAGCGTATGTCC

TGGTGGACTGTGGAGATAGCTCTGTATTTCTTGTGTAAGTATGAGGACAAG
CTTCGTACAGGCATAATCTGTGCAAATAAGGCAGCCATGGAGGCTAATGCT
TTTAAACTCTGTGCTAGGGAAGGGGAAGGCAGGCCAGTCCGTCTAGCTTG
CTTGGTGGCCCACAGTTAGTGAAAGGTATATGTGTGTGTGTGTGTGTGTGT
GTGTGTGTGTACAAGTATGTGCACCCCAGCACTCACATACAGGCCAGAGGC
AACTTTGTGAAGTCACTTTCCTCTTTCCACCTTTGTGTGGGTTCCAGGGTTG
GGACTCAGTTTAACAGGCTTGTGTGACCGGGTTCTGAAGTGTAAACTTATG
GTTTCTTTGCAAAGTTGATTGTGTAGGATGGTTTTTCACTAAGGCAGACACA
TGAAGGAGTGTTTAGCTGAAGCAGACACAGGTGAAAGGATGTTCTGCTAA
AGCAATCACGTGAAAGGATTCTTCCCTAAATGCCAGGATGTTCATTCACCTT
GCATTGTGCTACTGAGCTACGTTTGTCATGACTCTATGAAGAGAAACACAC
CAAAAACTTTCTGGCGGTGTTCTGGTGGCTGCTTGCCACGGACTTAGGCTG
ATTGGCAGAGTGATGTCAGCTGATACAGACTCACATGGAATTTTGCTAAGA
CAGACTCACGTGGTGAGGCAAGACCCATTGAACATCAAGTGATGTTTGGA
GGGAGTAGTAATAGAGCCCGATGGACAGCCATGGCAGGCTTGCGTAGCTAG
CTTTGCGACAGTTTGTGTTGGTCTCGTGTCTTTGCTGAGAGAGGCACAGCC
AAGAAGTTCACTTGGCATCCTTGCAGGTCCTTGGTCCCTCCTGCTGACTCA
TGCCGATTCAGGGGAGACCTGGCTTTCTCTGCTAGGTCTTGCCACTACTGC
CGATTTGTGTCTGCTATCCCGATACGACGGAACTGGCCTGCTGGTATATCCT
TGAAGGGTTTGCAAATGGACTGAGCTGCTGCCGCTGCTGACTCCTGTGAA
CTGAACTGCAGATTTCCTGACAACGCAGGTGGGATTTGCTCCAAAGAACA
ATTTCTAAGCGGGTCCACTTCTCCTTAACAACCTTTCTTTTCTACTACCTCTA
GTGGTAACAACCTTTCTTTTCTACTACCTCTGGTGGGTGGTGGGCTAGAAG
GGAGGTTAAATTGTTTAAGAAGCCTTATTAAAAGTAAGTCTTTGAAAAATCT
AAGCCTACAGGGCTCTACCCACTGAGCCGTCTCTCCAACCTCCTTTGGATAT
TTTTGGGCAGATATTTTGTCACAGAAGTGAGAAGAGTTACTAATGCAGTGC
CAAGCTAGACCTCTGCCTTCGACACACGTGTGCACACACACACACGTACTC
ACACACATCTGTACGCGCACCCCCTCACGTCCTCACCAGTATAACAGGCAC
ATGCACATTTACCAAGAATGTGTCTTCATGGTGGTCATTTTAGACCAACGCT
TATGCTCTTTAAATATTGGTTTTTAAATCTTTGTCACCTGAGGAATATTTTAAT
TAATTTCTTTAATTATGAAGTTTTTGTTTTTGGTAATATATCTACTGTCACTTA
TGAAAACCCTCAGGCTCCTGTTGTCTAATCCAGATCCTTTGTGACCATTTCA
ATACGTCATTATCTCTTTGTGTGTGTGTATGTGTGTGTGTGCATGCACATGTG
AACAGGTGTAGTCACTTGTATATGATTGTGTGGAGGTCAGGGGTTGACTTG
GGGTACTTTCCTCTCAATCTCTATTAATTAATTAATTAATTAATTAATTTCTCT
GTGTGTATAGACATGTGTATGGGTATATAAGTAGTGATGTTGGGTATCTTCTT
CTATTTTATTTTACTGTCTTTCTTTCTTTCTTTCTTTCTTTCTTTCTTTCTTTCT
TTCTTCCTTCCTTCCTTCCTTCTTTCTTAAAGGCTTATTTATTTATTTATTATAA

GTACACTGTATCTATCTTCAGACACAACAGAGGGCGTCAGATATCATTACAG
ATGGTTGTGAGCCACCATGCGGTTGCTGGGATTTGAACTGAAAGAGCAGTC
AGTGCTCTTAACCTCTGAGCCATCTTTCCAGCCCCTCTTTCTTTCCCCTCTTT
CTTCTTCCTTCCTTTCTCTCTCTTTCTCTCCCTCTGTCTCTCTGTCTCTCCCT
CTCTGGCTTGGCTTTTATTTGGATTCTAAATATCCAAATAAAAGAGCCAAAT
TCCAGTCTTGGTGCCTGCACAGCAAGCACTTTGCCCCCTAAGCCATCTCTC
CACTGTGGCCACATTAAGTTTTGAAAGAGAGTTTCTTAAGTGAACCCAGAA
CTCATGTTTCAGCGAAACTGATTGAACGATGAGACCTCAGGACCCTCCTGT
CTCTGCCTTCCAACACCAAGGTTACAGCTGTGTACCACATCTGCCCATATAT
CGGTGTTGAGGATCCAAACATAGGTCTGATGTTTGCATATGAAGCACTTTAT
ACATGTAGCCATTTACCTGACCCTGTCCCATTATCTTTTTTTTCCATTTTTTTA
TTAGATATTTTCTTCATTTACATTTCAAATGTTATCCCCAAAGCCCCCTATACC
CATCACCACCCTGCTCCCCAACTCACCCACTCCTGGTTCCTGGCCCTGGCA
TTCCCCTCTACTGGGGCATATAAAGTTTGCAAGACCAAGGGGCCTCTCTTC
CCATTGATGTTTTTTTTTTCTTTTAATTAAAAAAATTTTTTTAAATTTTTTATAT
TTTTTCCCATTATCTTTTAAACCATTTCCTGCTATTTATTTCTTTTATGTGTAA
AGCATTTTCTTATTTTTTTTTCTATTTCTTTAAGGGATTTTTATAATTCTTCCCT
TCTTGCTTTCATTAGAGATGTCTTTATTTCCAAAATAATTTTTCTTCTTATTTT
AGATCTTTTCTTGATTCTTGTCACTTCATTTCTAGGTAATTCGAATTTTAACT
TGCTTTCATAGCTAATTGTGTATTGAGGTTTGGTCTGTTGCTATGTATTGTAA
TGTTAAATATTGGCCCTGGCTGCCCCTAGGGATGAGGGATTCTCATGTAGAC
CAACTGATGCTATGTAACCTTGCTCCCCAAGTTATCCCTGATTGGTGAATAA
AGGTGCCTACATCCTATGGCAGAAGAGATGTAGGCAGAGTTTTGGTTCCTG
GGCTTTGGGGCTGAGGTGGAGATCAAAAGGAGAGAAGAGAAGAAGGTGG
AGAGAGGAGAAGACAACATGGGGTAGGTGAATCATGACAACATGGCCATG
AGAGCTGGCCAGTTGGAGCTAAGAGCAGCCCAGACAATACATGGCTAGTC
ATATCTCGAGGTTATTGTTAGGGAAGTAGACATAATAGTATAGAGGGTAGAT
ATCTGTTCAGCACCAGTACTTGGAAGGGTTATTATAAATATACAGGTTTTGT
GTCTTTTATCTGGGAGCTAAGTGATTCAGGGCACAGTAGAAACCCCTAATT
GAAATAAAATATTTACGACAACAGTTGTGTTAGTTTATTTTTTACTTTTAAAA
TAGAAACTTATAGGACCCCCCTCCCCCCCAAAAAAGACCAGGTCATTTTAG
CATACTTTTATTGTGTATCAGTTACATATTGAATCTATGGGGTTTATTAGCCAT
CACCATTTGTTTCTATTTTTCATGCTTTGACAGTTTCGTGGAAGTCCATAATG
AATTTTAGTCATTTTCTTTCTGTTGTTTGTCTCTTGTGCTCGTGGCCATCAGA
TGGTGCAGTGTTGCACACTGCTCACTGATAGGCAAGCCTTCAGGCAGTTTG
TCCTTGTTCAGTTTGTATCTTGTGAGAATAACGTGCCACGCCACAGTGCATC
ATCTCAAACCCTGCCTCCTCTTTTCCTGTTCTCTGCATTCTGTTCTGACCTTC
AGACCCTGAGGTAGGGCCCGAGTTCGCTACTGAATCTGAGGTAGCTCTCTG

ACGGATGTGACCAATGGAACGTGGAGAAAATGATATCCTGGGTGGCTGTCA
GGGCCCTGGCATCATGTGATGAGGATACCAAGCTATTCAGCCAGGTCGCAG
CCAGAGCGCAGTGACAGTTTCAGCTGAGCTCCCAGGCAATGATCCTGCCT
GAGCTCCCAGGCAACCGTCCTATGAAGGAGGACCCTGAACGTCTATCCAGT
CAAACCTCCTGAGGACTTCAGGCTGCTCTGTAAGAGGTGGTGAACAATAG
TTGTTTTAAAGTTACAAAGTACAAACTACAAATTATCGGTTGAGGTTTTGAG
GAAGCAATAGATAATCAACTACGAGATTCGTTTCAGCTGTTGTCTCCTTTTG
ACTTTGCTATCGAGTTGGTCCTTGCCTTGGGTGTGGGAATTTGTAGAGTCTA
CAGCAATGCCCTCCTCCCAACCCTTTCCCAGAATCCACTCTCACATGAGATT
ATAAGGTTCATTTAGGATAGACCTCAGGCAGGGCCGTGGAGCCACCAGAG
AGTCTAATTCAGAACCAGAACAGAACACGTGTCCTTTAGAGCTCCTCCCCA
TGGACGGTGGCCTTCTGACCAACTCTGCATGAAAATAGCCTCCTCCTCTTC
ATATCCTGCCCCGCCGCCGCCCAAGTTTACGCTTACATTGTATAAATCTAATA
ATTTACATATTTGAAAATGTTTTCCAGGCTGTTATATCTAAAAAGGCAAGTG
AGTCATGCAAGTGTTTCAGTCAAGGTAATTGTATACCCCAATCCATCCATTC
ATACAAAAATATATTTACTGAGGACTTATGGTGACCCAAGAATTGTCCCTGC
CATTAGGGGCATTGTGGTATTGTGTGTACTTCACTTAACAAAACAAGCATCC
TTACCCTTGGGGAAATTAAAGCCTGGTGCTGTATATTATATCATTGAAGCGTT
TATTTAGTGGTATGCGATTGTTACTTGAGTATGCTCCTGAACAAGTGCCAAA
CCCACTTCCAGAACCCTCAGCCAACTTCAGCACACTCCTCAGAAAGACTCT
AGCAAACAGTGTTTTCATTTCGGTTCTGGTGATGGAGTAGACGCTCTTGGC
AATACTTCTCTGGATGTGATCCACCTAGTTAGCATTCCTCTAATTCAGTTCTG
ATTTCAAAAATTACTAAAATGTGCTAAGTAGTTTCAGGGGATAATGGAGCAT
TATTTTGTTGTTGTTGTTGTTAAAAATAAAAGTTACCGAAGGAGGCACTTTA
ACATTTCCTGTTCACATGGGATCGTGAGAATGGCACTTCCTTAGATCAGTAA
GAATTTGGAGTCTTTGTGTATAGATGGGGTAAGGGGTGTTTAGTAGAGACT
GTTACAAGAGGAAGAGAAGAAATACAAGGTCATATTCTCAACACTAACAA
CTTAGGAAAATGTAAACTTTTAGTAGCTTCCCTCACACACCACCTGGAAAT
AAGCACCAGCAGGAAGGTCTCTACCCGCCTCTGACTCGCCTTTCCCATCCC
TGGGTCTTCTGGGATCTGTTAGTGGGCGGGAGACATGGTCATTGTTTATAAT
GCTCACAAGCCTGCATGGATGCAAAAGATATATATTTAGAAAGCAAAATAA
AGCTTTAGGAAGATCTTCAATTTAATGGAAACTCGTCTAGTCATTTGAGAAC
CACAAGAGCTTTTTAGAGAAGGCCCACTTCTGCCAGCTGGAGGAAGTGTG
GGTGTATGGGGTCCCGGTGACACCTGTGATGTCAGTCACCCCGAGCCTGAG
GCTCATTGGCTCTGAGGAGCTGCAGCAGAGTTTAAATTCTGCACTGTCAGA
CAAGCCTTCTGAGCAGCAGCTGGAGAGTGGCTTCTCTGAGATCCCTCTTCG
AGAACTTCGCTGAGGTTTTGACCTTGTGGGAAAAAGACCTTGGGAGCCAG
AGAGGGTAAGGCCATTCCATCATTTAATTCTTAAAATGACTTTAGCTTTGGA

GTTTAAAAATATGAAATACGAACATGTTACAAGGCATTGATTTCCAGAGGA
AGATGGGGAAGAGCTAAGTTTGAGTAAAACCTATTCTTATTTTTGTTTTTTG
GGGTCACTGTCACAATAGCCAATGACACTGATATTAAAAATGACATGGTAA
GTCAAAAGAGCCAGGTGTTTGCCTGGTGATGCCTTAATTAGATCTCTGTCTA
ATTATTTTGAAAGAGAACATTTACTAAATAGGAGGCTGATGGGAAAGTCCT
GTTAGAGTTTAGCTTTCATTAATAAAACACAACTTTTATCTGTAGGCTTCTTA
GAGGTGGTGATGCGAAAGTATCTAAATCAGTGTAGCAAGTGTTTCAAAATG
CAGACAGGTAACTGAATGCCTCTCAGCCTGGCTTATTCAGAGAATACGGTG
GCCATTATGAATTGTGTATACAACTGAAAGGTGATCATGACAGTGTGGCTTT
GGGTAGCTGGATCCTCATGGTAGTATAATGCTTTGCATGCAGATGGACTCCA
ATAAATGCTGAGTGGGATTACCTGTTAGGTAGGACACACACATACTACACA
CACATATACATACATACATACATACATACATACATACATACATACAAACAAAC
ATACACACACATACATTAGGTAGGACACACATACACATATCACACACACCAC
ACATACATACACACACATAGATGCACATGCACATACATTAGGTAGGAGACAC
ACATACATACACATACCACACACACACACACACACACCTCGTACCATATA
CACATATACATTAGGTAGGACATACACACACACCTATACACAGATATATTAGG
TAGGACACACACACACACATACACACCTACATTACATAGGACACACATATA
CATTAGGTAGGAGATACATAAATACTACACACACATACACTCCCACCAGACA
TGATTTTACTCTGCATTACTTCACTTCATCAGTAGCTGGAATATCTATCCTTC
TGACAGAGAGTAGCCAGGCCATGTGTCCTACGAAGTTCTGAAGACACCGA
ATGGAGAAAGTGAATTGTCCTCTGTGAGAGCAAAGACTCCTGGTTTCCTCA
GGTTTCCGTTTGATTGACACTCTTGTGGCCTGCCTCTATTTAACCCTGTTGA
TTAAACACATGAGAACAAAGAATTTTCTGCAGATTCTTTAGTCTGTTTACTG
TTCACCAAATGGTGATGTGGACCAGGGTGTCTCAGAAGTTTAGAGGTTTCT
ACAACCTGTATTTGGGGCACTTTGTGATTATCCTGCTGTGGAGGAAGGAGT
ATAATACTTGGGTTGGTAGCAATTGTCCTAGAGACACTCACAATTACTGGAA
AGATGGGAAAGCTTTCTTTGTTCATTTAGTTCTCTGAATTTTAATAGAGTTT
GTATTCTGAGCAAGTTGCACTTTATTTTCAAAGAGTAATGGTGAAGAAATTA
AAACAATTGCTGATTAGCTGATTATAGTAATAACACTATCATTTATTATTTTTA
TAGGGTAAGTATTATATCTCAGCACTTAGAACAGTCCCTAACATATTGGATGT
TCTCAATAAATAATTATTAGTGACTCATAAATGAATATGTTATTCATTAATATT
TTCAAGGTTGACAATTAGAAGAAAAATCGAAGTTATTTTCCAAGGATTTAA
AAAAAGAGCCTATGACTTTTTTCTCCCTTTATTTCAGATTACATCAAGAAGA
TATATATATATATATATATATATATATATATATATATATATATATATATATATATA
CCTTACGAGTGAAACTTAGCTTTGAGGAGATGCTAAAACAAGTTTCTTACT
TAACATAGAGTTTTGAACAAATTTGGAAACACAGCCCTTTCCAGATTCTGA
CAATTTAGATCTTTTTCAATTCCAGGTTAACCACCCAGAAGGGCTGGATGTC
TCTAGGGTAGTGACTTCTGAGCAGTCATGATTCAAGTCATGGCTGTTGAGT

TCCATCACTTGGATGCTCTTATAAAACAACCACATATCAAAGGTAGAAATTC
TATAATCAGTTATGGAAGGAGAGCTTGTGGCTCTGGCTTCATGGTATGTAGG
CCTGGAGCTACCTTGTTAGCCTGATAATCAGAATTCTGAGTTGATTTCTGCT
TCTAATCTTTCTTGAAGAGTAGCTCAAAAGTTTGGTGATTTCATTTGGAAAT
ATTCTTCACAAAAACATCAATATCACAACAAAAATAATAATTGAAAACCCA
ACATAGGCTTTAGGGTGTGACCTTGTTCGGTGGGGCTATATTCTCTGAGAGC
CGACATCAGCTCTCTTGTGACGCTAGGTTGTCTTGGTTTTGGCCTCCTTGTC
TTCTATTTGGGAAATTACTGTTTTAGGTTGGGATGTAAATGGTATTTCTAGAA
GCCTGAGCATGTTAGGTCATCTTCCTTACTCTGCTCCCTATTCTACTCCTGAG
AAACTTGAGCAATGACATCATACACCTCCTGGTGAGAAACCCATAACCTTT
ATTGGTTTACAGTAGCTGCATATTTTACCTCCACACTTTTCAGTTATTAGGTA
GTAGAGAAATTAAAGAAGATGCAAGAGAACTGAGGAATCACTGGTGTTTT
CTGGGGGATGTTTCATAGAGTTATGAAGTTACACCTCTACTTAAACTGTAAA
AATCAGAAAATTGTAAAATGCAGGAAATCTGCACAACGTTCCTTTTGCCCG
TGAAACCAAAGCAACTGACATGTAGTTATTCTGAGAAAATTGCATCTGAGA
ATTTTTTCAACCTTTTAAAAAGAATGAATCTAAGAACTTAAAAACTGTTTGG
TTGTAAGAAGCGACATCTGTTGAATTAGACTTATTTTAGCTACAGTTGCATG
TCTTCTAATATGGTCTTCCCAGTTGATATTATGGAAAGAACTTAAAAATAAAT
CAACATAATATGAAAACTACTTATTGTAGCATGTTAAATTATAGTAAAGAAA
ATATTTTGAAAGATTCCCAAGTGGTCCTAATAGATATCCCATGTTGCAAACC
TGTGGCTTAAATGTATTTTCACTATCTGTCGTGCATTTCCATAGAAACAAAG
AAACTGATGAGTACAGAATGGGAACTGAACTTGCAGAATTTCTTAAAACAT
GTGGAGTTCAATCAAGTGCGCCTGTTAGAGCAGCTGTGAGGGTTTACCAG
AGTCGGGAAATTATTTCCCTTCAGACCATTTTGATAGCACATCAGCATTGAG
TGGGTCCACATTGAGATAAAGAACTGATTATTTTTAAAAGCTGCTGGCTCAC
AAAGTTTGGAACAAAGCAACCTCCCAGAGGAGTACAGCTTGAGGTGGCCT
GTTCATCTCAGTGTGACTCACAAGTGTTTCCCTAACCTCTAATCTTCTCTGG
GTCCTCCGGTGCCGTGAATGACACTTGAGAGAATTTAGCACAATCACGTAT
AATGTATGGCTGAATGCATCACGGTAGGCATTAAGAGCTTTCTGAGGCTGTA
ATATGAAGCCTGCCACAGCACGTGGCTTGAAGATCAAAACGAAAAAAGTG
CAAAATCAAGTTATTTTTGTCTAGACTTTATTATATAGCTATAAAAATGACTG
AAGACACACACACAGACACACACACACACATGTGCACACACAGGGGTTAG
TTTTAGGAGAATAAAATGTTAGGTTGCTGTGTAATACTGGCAATGTGTCTGT
TCCCTTTTCTTTAACCCCTTTCTATTTTCAGCTTCTGAGTTGGTGGAGAGATA
CCTGTAGGCCTCCTCTTTAAGATTTCGTTTCTCAGGAGTGATGTTTCTGTTG
ACTTTTTGAACGGCTTTAGGAAGTGTAATTTACATGGCACTTTCCTATGCGA
AGCATCTAGTGGAGTGGTTTTTGCACACTCACTGGTGTGAACAACATTTAC
CACAATCCCTTCAAGACATCTTCATCACCTACAAAAGACATCTGCACCTCC

ACCCTCACTTCTCAGCCCCTGCCCCCTGTAACTCCAAGCCCTGAAAATCAC
AGATCTGCTTCCTGTCTCTAAAGATTTGCACATTCTGACCAACTTATAGAAA
TTAAGCCATACAACTGAGGTCTTTTATACCTGGCTTCTTTTTAACCTGTATGA
TGCTGACAAGGCTCCTCTCCTTTGTGGCATCTCTTGGCGTTTTGTTCTAGTT
TTATGGCTGGATGCTATTCCATCCTACAAATGCAGTCTGTATTCTTCGCTCTT
TCACCCACATGTTCAGGTGAACATATTTATGGTTTTTTTTTAATTAAAAATGT
AAAAATGAAAGCATCCTGATTACTGTATCATGAAGTGAAACATCTACGACTT
TCATTACAGGTAGAGGAATCGCATCCCAAT
