# Supplementary material for: Identification and Mechanisms of Osteocyte Subsets Involved in the Pathological Progression of Osteoporosis
Source: Adv Sci (Weinh). 2025 Nov 18;13(5):e13427. doi: 10.1002/advs.202513427 (PMC12850396; doi:10.1002/advs.202513427)
Supplement: Supplementary file 6 — Supporting Information [file ADVS-13-e13427-s004.zip › Supplementary File 2.pdf]

# Name	
# Sequenc	CTGAGCCAACAGTGGTAGTAAGG
# Genome	GCA_001632555.1
# PAM	NGG
# Position	CM004225.1:155338-155361:+
# Version	CRISPOR 4.98, 2021-03-01T04:09:08CET
# Results	http://crispor.org/crispor.py?batchId=TGCivxyF5X1XeiqiPAeK

guideId	guideSeq	offtargetSe	mismatchP	mismatchC	mitOfftarg	cfdOfftarg	chrom	start
21forw	CTGAGCCA	CAGAGCC	.*...........**..	4	0.03215	0.553633	CM004219	1.08E+08
21forw	CTGAGCCA	GTGACTTA	*...***.........	4	0.564015	0.541246	CM004224	44011833
21forw	CTGAGCCA	ATGAACC	*...*......*.....*	4	0.152684	0.539259	CM004218	27548679

end	strand	locusDesc
1.08E+08	-	CM004219.1 107.62 M
44011855	-	CM004224.1 44.01 Mb
27548701	+	CM004218.1 27.55 Mb
